# Nucleophagy delays aging and preserves germline immortality

**DOI:** 10.1038/s43587-022-00327-4

**Published:** 2022-12-23

**Authors:** Margarita-Elena Papandreou, Georgios Konstantinidis, Nektarios Tavernarakis

**Affiliations:** 1 Institute of Molecular Biology and Biotechnology, Foundation for Research and Technology-Hellas, Heraklion, Greece; 2 Department of Basic Sciences, School of Medicine, University of Crete, Heraklion, Greece

**Keywords:** Autophagy, Germline development, Ageing

## Abstract

Marked alterations in nuclear ultrastructure are a universal hallmark of aging, progeroid syndromes and other age-related pathologies. Here we show that autophagy of nuclear proteins is an important determinant of fertility and aging. Impairment of nucleophagy diminishes stress resistance, germline immortality and longevity. We found that the nematode *Caenorhabditis elegans* nuclear envelope anchor protein, nuclear anchorage protein 1 (ANC-1) and its mammalian ortholog nesprin-2 are cleared out by autophagy and restrict nucleolar size, a biomarker of aging. We further uncovered a germline immortality assurance mechanism, which involves nucleolar degradation at the most proximal oocyte by ANC-1 and key autophagic components. Perturbation of this clearance pathway causes tumor-like structures in *C. elegans*, and genetic ablation of *nesprin-2* causes ovarian carcinomas in mice. Thus, autophagic recycling of nuclear components is a conserved soma longevity and germline immortality mechanism that promotes youthfulness and delays aging under conditions of stress.

## Abstract (web summary)

Nuclear morphology changes with aging, but the role of these changes and the underlying mechanisms are not fully understood. The authors find that the nuclear envelope anchor protein ANC-1 in worms, and its counterpart nesprin-1 and nesprin-2 in mammals, promotes the degradation of nuclear components to limit nucleolar size and function in a soma longevity and germline immortality mechanism.

## Main

Gradual deterioration of nuclear architecture is a common denominator of aging and many age-related pathologies in diverse species, including humans^1–6^. Notably, progeroid syndromes and aging itself are characterized by substantial expansion of the nucleolus, the largest well-defined structure within the nucleus and the site of ribosome biogenesis^7,8^. By contrast, longevity and life span-extending interventions have been associated with small nucleolar size^9^. Nucleolar size is a highly predictive marker for wild-type (WT) *Caenorhabditis elegans* longevity^9^. However, the mechanism(s) that bring about these marked changes are obscure. Moreover, the contribution of major pathways that modulate germline immortality toward shaping the nucleus during aging is not understood. To uncover the cellular and molecular underpinnings of nuclear morphology breakdown during aging, we examined the involvement of nuclear material-selective autophagy (nucleophagy)^10,11^ in the preservation of nuclear and nucleolar homeostasis^12^. Previous studies have implicated two nuclear envelope, spectrin-repeat proteins, nesprin-1 and nesprin-2, as determinants of nuclear size in eukaryotic cells^13,14^.

In this study, we examine the mechanistic and physiological significance of degradation of nuclear components. We unveil that nucleophagy is a crucial element of germline immortality and somatic aging. Moreover, dampening of autophagic recycling of nuclear membrane and nucleolar components eliminates resistance to DNA damage, nutrient and heat stress. We reveal that abnormal nuclear anchorage protein 1 (ANC-1) and nesprin-2, which are degraded by autophagy, are key nucleophagy regulators and restrict nucleolar size, a common denominator of diverse life span extension regimes. Moreover, we show that ANC-1/nesprin-2 ablation leads to infertility and formation of tumorous structures in the gonads and ovaries of the nematode and mouse, respectively. Ultimately, nucleophagy confers stress resistance, germline immortality and longevity.

## Results

### Autophagy modulates nuclear architecture via ANC-1/nesprin-2

**Fig. 1:**
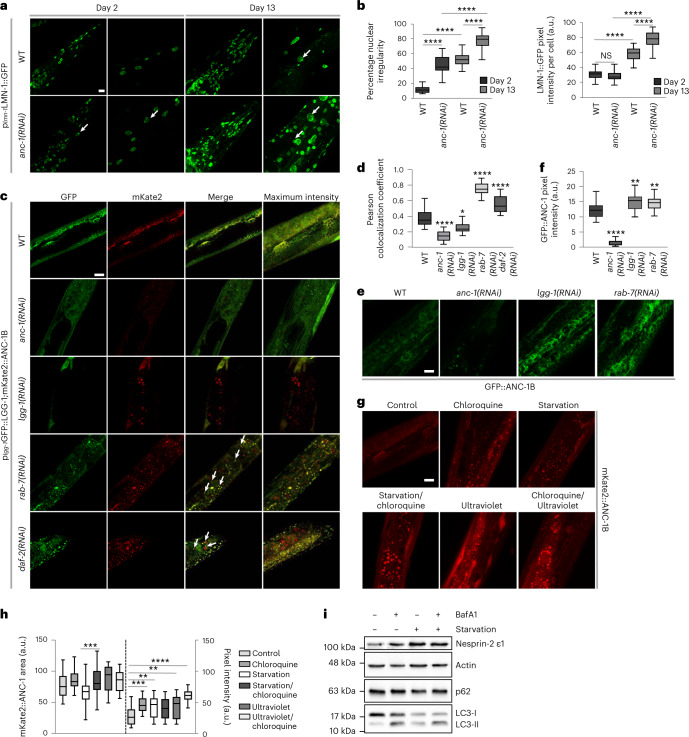
Autophagy targets the nuclear membrane proteins nesprin-1 and nesprin-2. **a**, Confocal microscopy imaging of control (WT) and *anc-1(RNAi)*-mediated silenced worms of the indicated age (day 2 and day 13), expressing the p_*lmn-1*_LMN-1::GFP reporter. The arrows indicate irregular nuclei (micronuclei, irregular shape, LMN-1 aggregation). Scale bar, 20 μm. **b**, Quantification of irregular nuclei per worm head and LMN-1::GFP fluorescence intensity in *C. elegans* head and body from **a**. The box plot represents the 25th–75th percentiles, the line depicts the median and the whiskers show the min–max values. NS ≥ 0.05, ****P < 0.0001 using a two-way ANOVA; *n* ≥ 18 worm heads. **c**, Confocal microscopy imaging of day 2 adult worms expressing p_*lgg-1*_GFP::LGG-1 and mKate2::ANC-1B in WT worms and on RNAi-mediated silencing of *anc-1*, *lgg-1*, *rab-7* and *daf-2*. The arrows indicate GFP and mKate colocalization. Scale bar, 10 μm. **d**, Colocalization analysis (Pearson colocalization coefficient) between GFP::LGG-1 and mKate2::ANC-1B from **c**. The box plot represents the 25th–75th percentiles, the line depicts the median and the whiskers show the min–max values. *P < 0.05, ****P < 0.0001 using a one-way ANOVA; *n* ≥ 18 worm midbody areas. **e**, Confocal microscopy imaging of day 2 adult worms expressing GFP::ANC-1B under *anc-1*, *lgg-1* and *rab-7(RNAi)*. Scale bar, 10 μm. **f**, Quantification of GFP::ANC-1 fluorescence intensity from **e**. The box plot represents the 25th–75th percentiles, the line depicts the median and the whiskers show the min–max values. **P < 0.01, ****P < 0.0001 using a one*-*way ANOVA; *n* ≥ 27 worm midbody areas. **g**, Confocal microscopy imaging of day 2 adult worms expressing mKate2::ANC-1B after chloroquine, starvation, ultraviolet or combined treatments. Scale bar, 10 μm. **h**, Quantification of mKate2::ANC-1 fluorescent area and intensity from **g**. The box plot represents the 25th–75th percentiles, the line depicts the median and the whiskers show the min–max values. **P < 0.01, ***P < 0.001, ****P < 0.0001 using a one-way ANOVA; *n* = 25 worm midbody areas. **i**, Western blot analysis of the nesprin-2 isoform ε1, p62 and LC3 from E14.5 MEF lysates under control, BafA1, starvation and BafA1/starvation conditions. Source data

**Extended Data Fig. 1:**
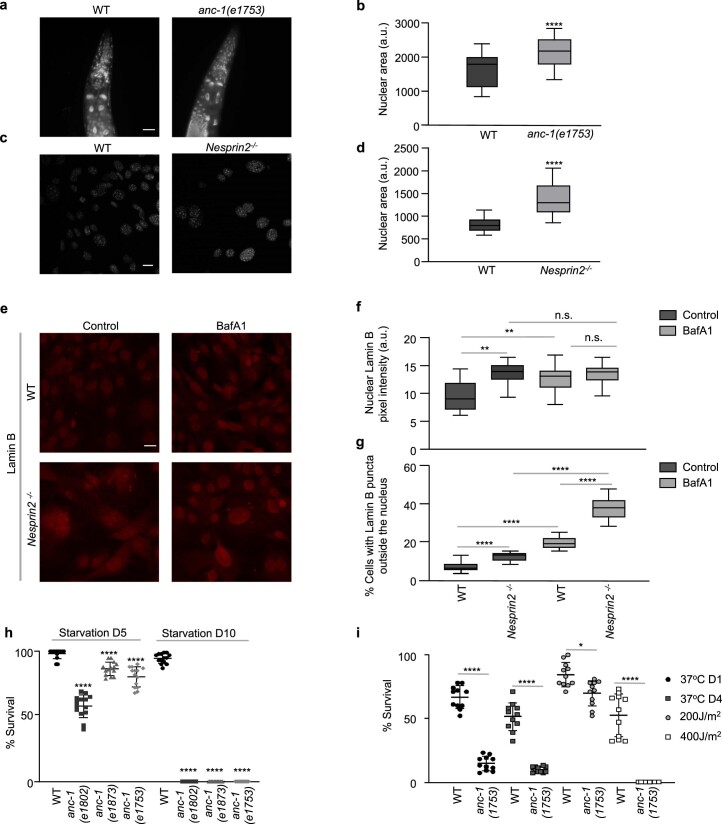
ANC-1/Nesprin 2 regulates nuclear architecture. **a**, Epifluorescence microscopy imaging of day 2 worms stained with DAPI. Scale bar, 20μm. **b**, Quantification of nuclear area from **a**. The box plot represents the 25th–75th percentiles, the line depicts the median and the whiskers show the min–max values. ****P<0.0001 using unpaired *t*-test; n = 41 nuclei. **c**, Confocal microscopy imaging of WT and *Nesprin 2*^*-/-*^ E14.5 MEFs, stained with DAPI. Scale bar, 20μm. **d**, Quantification of nuclear area from **c**. The box plot represents the 25th–75th percentiles, the line depicts the median and the whiskers show the min–max values. ****P<0.0001 using unpaired *t*-test; n = 41 nuclei. **e**, Epifluorescence microscopy imaging of Control and BafA1-treated WT and *Nesprin2*^*-/-*^ E14.5 MEFs, immunostained for endogenous lamin B. Scale bar, 20μm. **f**, Quantification of nuclear lamin B pixel intensity from **e**. The box plot represents the 25th–75th percentiles, the line depicts the median and the whiskers show the min–max values. n.s.≥0.05, **<0.01 using one-way ANOVA; n = 34 cells. **g**, Quantification of percentage of cells with lamin B puncta outside the nucleus from **e**. The box plot represents the 25–75 percentiles, the line depicts the median and the whiskers show the min–max values. ****P<0.0001 using one-way ANOVA; n ≥ 21 cells. **h**, Percentage of survival of WT, *anc-1(e1802)*, *anc-1(e1873)* and *anc-1(1753)* worms after five or ten days of starvation. Mean ± s.d. ****P<0.0001 using two-way ANOVA; n = 14 worm survival assays. **i**, Percentage of survival of WT and *anc-1(1753)* worms after acute heat stress (37 °C) at day 1 or 4 of adulthood and UV-C induced DNA damage at day 1 of adulthood. Mean ± s.d. *P<0.05, ****P<0.0001 using one-way ANOVA; n = 11 worm survival assays. Source data

**Extended Data Fig. 2:**
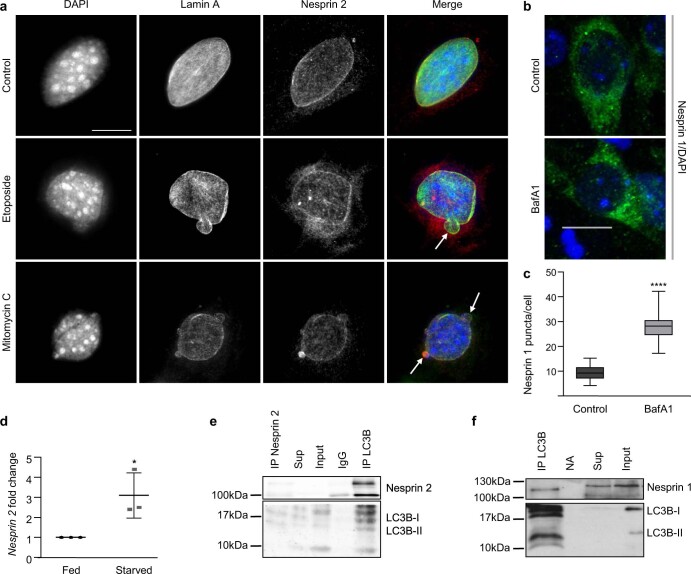
ANC-1/Nesprin 2 localisation upon stress. **a**, Epifluorescence microscopy imaging of WT E14.5 MEFs in control, etoposide- and mitomycin C-treated conditions stained with DAPI (blue) and immunostained for endogenouslamin A (green) and nesprin-2 (red). Scale bar, 20μm. **b**, Confocal microscopy imaging of WT E16.5 cortical neurons stained with DAPI (blue) and immunostained for endogenous nesprin-1 (green). Scale bar, 20μm. **c**, Quantification of puncta number per cell from **b**. The box plot represents the 25th–75th percentiles, the line depicts the median and the whiskers show the min–max values. ****P<0.0001 using unpaired *t*-test; n = 49 cells. **d**, Quantification of *nesprin-2* expression levels by qPCR in WT fed and starved (EBSS) E14.5 MEFs normalised to the *HMBS*. Mean ± s.d. of three biological repeats. *P<0.05 using one-way ANOVA. **e**, Immunoprecipitation of nesprin-2 from mouse kidney, followed by western blot for nesprin-2 and LC3B. **f**, Immunoprecipitation of nesprin-1 from mouse cortex, followed by western blot for nesprin-1 and LC3B. sup: IP supernatant, IgG: mouse IgG antibody control, NA: no antibody control. Source data

We investigated whether the *C. elegans* nesprin ortholog, ANC-1, regulates nuclear morphology during aging. Pronounced nuclear enlargement, shape abnormalities and lamin-1 (LMN-1) accumulation occur in the absence of ANC-1/nesprin-2, compared to WT animals and mouse cells (Fig. 1a,b and Extended Data Fig. 1a–d). Lamin B1 is a substrate of autophagy on oncogenic insult and exhibit increased protein levels in the absence of *nesprin-2*^15,16^. Depletion of nesprin-2 causes lamin B accumulation in mouse embryonic fibroblasts (MEFs), which is mediated by blocking autophagy. Indeed, treatment with the autophagic flux inhibitor bafilomycin A1 (BafA1) increases lamin B abundance only in WT cells, while abnormal lamin B staining outside the nucleus is prominent in the absence of nesprin-2 (Extended Data Fig. 1e–g). Therefore, autophagic recycling of lamin B is, at least partially, regulated by nesprin-2. Thus, the role of nesprin family members in maintaining nuclear architecture is evolutionarily conserved from nematodes to mammals. Importantly, ANC-1 is essential and confers stress resistance against nutrient deprivation, heat and DNA damage (Extended Data Fig. 1h,i). Since autophagy is triggered as a cellular response mechanism to various stressors, we hypothesized that nesprin family members might be engaged in a selective type of nuclear autophagy to ultimately contribute to nuclear morphology homeostasis^15,17^ and stress resistance. To test this hypothesis, we first examined whether ANC-1 colocalizes with LGG-1 (microtubule associated protein 1 light chain 3 (LC3), gamma-aminobutyric acid receptor-associated protein (GABARAP) and GATE-16 family-1), the nematode autophagy-related protein 8/GABARAP protein homolog, on autophagy induction and autophagic flux inhibition by knockdown of insulin-like receptor subunit beta (*daf-2*) and Ras-related protein Rab-7 (*rab-7*), respectively (Fig.1c,d). Moreover, genetic inhibition of autophagy by *lgg-1* and *rab-7* RNA interference (RNAi) leads to accumulation of green fluorescent protein (GFP)::ANC-1 (Fig.1e,f). Similarly, autophagy-inducing conditions such as starvation and DNA damage (ultraviolet) modulate ANC-1 expression and nuclear area, as shown by using the mKate2::ANC-1^18^ reporter (Fig. 1g,h). In MEFs, nesprin-2 localization is also altered on autophagic induction by DNA damage (Extended Data Fig. 2a), while nesprin-2-decorated nuclear protrusions form under DNA damaging conditions (etoposide and mitomycin C). Interestingly, these protrusions are analogous to the nuclear bulges formed during nucleophagic degradation of nuclear material^15^. In cortical neurons, nesprin-1 distribution is more punctate on autophagic flux inhibition (Extended Data Fig. 2b,c). On autophagic flux inhibition, the relative protein levels of the nesprin-2 ε1 isoform are elevated (Fig.1i), indicating that nesprin-2 is degraded via autophagy. Notably, we find that *nesprin-2* expression increases under autophagy-inducing, starvation conditions as shown by monitoring relative RNA and protein levels (Extended Data Fig. 2d and Fig.1i). In addition, immunoprecipitation experiments indicate LC3 interaction with nesprin-1 and nesprin-2 (Extended Data Fig. 2e,f). Taken together, these findings suggest that nesprin-2 abundance is regulated by autophagy.

### ANC-1/nesprin-2 regulates autophagosome formation

**Fig. 2:**
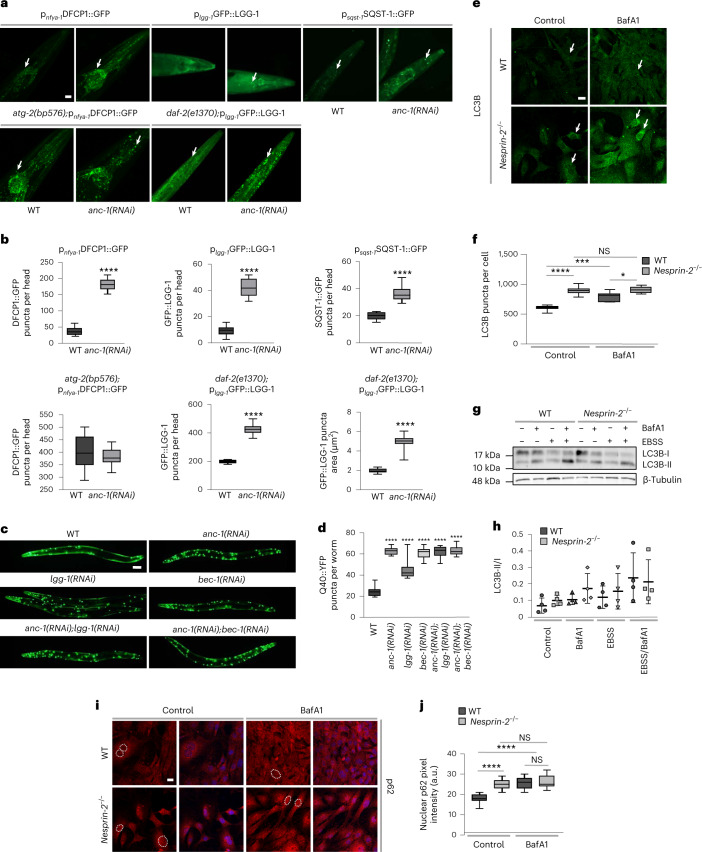
ANC-1 and nesprin-2 regulate autophagy. **a**, Epifluorescence microscopy head region imaging of day 2 WT worms and *atg-2(bp576)* and *daf-2(1370)* mutants, expressing the indicated reporter genes, subjected to RNAi-mediated silencing of the *anc-1* gene. The arrows indicate punctate structures. Scale bar, 20 μm. **b**, Quantification of GFP puncta per head and puncta area from **a**. The box plot represents the 25th–75th percentiles, the line depicts the median and the whiskers show the min–max values. ****P < 0.0001 using an unpaired *t*-test; *n* ≥ 17 worm head regions. **c**, Epifluorescence microscopy imaging of day 1 p_*unc-54*_Q40**:**:YFP worms on RNAi-mediated silencing of the indicated genes. Scale bar, 20 μm. **d**, Quantification of Q40::YFP puncta per worm from **c**. The box plot represents the 25th–75th percentiles, the line depicts the median and the whiskers show the min–max values. ****P < 0.0001 using a one-way ANOVA; *n* ≥ 29 worms. **e**, Confocal microscopy immunofluorescence imaging of LC3B in control and BafA1-treated, WT and *nesprin-2*^−/−^ E14.5 MEFs. Scale bar, 20 μm. **f**, Quantification of LC3B puncta number per cell from **e**. The box plot represents the 25th–75th percentiles, the line depicts the median and the whiskers show the min–max values. NS ≥ 0.05, *P < 0.05, ***P < 0.001, ****P < 0.0001 using a two-way ANOVA; *n* ≥ 22 cells. **g**, Western blot analysis of endogenous LC3B in control and Earle’s balanced salt solution-starved WT and *nesprin-2*^−/−^ E14.5 MEFs, with or without BafA1 treatment. **h**, Quantification of the LC3B-II/I ratio. Mean ± s.d. of four biological replicates. **i**, Confocal microscopy imaging of control and BafA1-treated, WT and *nesprin-2*^−/−^ E14.5 MEFs stained with DAPI (blue) and immunostained for p62 (red). The dashed lines represent nuclei. Scale bar, 20 μm. **j**, Quantification of nuclear p62 fluorescence intensity in control and BafA1-treated, E14.5 MEFs from **i**. The box plot represents the 25th–75th percentiles, the line depicts the median and the whiskers show the min–max values. NS ≥ 0.05, ****P < 0.0001 using a two-way ANOVA; *n* = 19 cell nuclei. Source data

**Extended Data Fig. 3:**
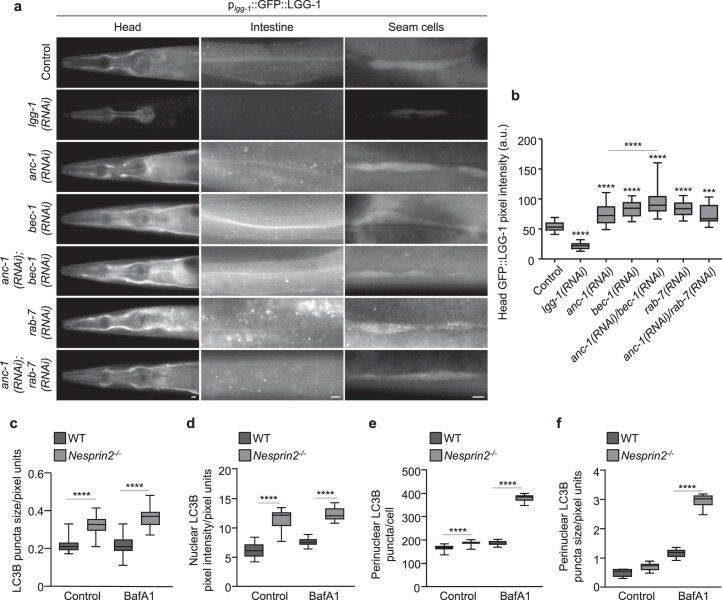
ANC-1/Nesprin 2 regulates LC3B abundance and localisation. **a**, Epifluorescence microscopy imaging of head, intestine and seam cells of GFP::LGG-1 transgenic worms subjected to RNAi-mediated silencing of *lgg-1*, *anc-1*, *bec-1*, *rab-7*, *anc-1;bec-1* and *anc-1;rab-7*. Scale bars, 20μm. **b**, Quantification of GFP::LGG-1 pixel intensity in head from **a**. The box plot represents the 25th–75th percentiles, the line depicts the median and the whiskers show the min–max values. ***P<0.001, ****P<0.0001 using one-way ANOVA; n ≥ 20 worm head regions. **c, d, e, f**, Quantification of LC3 puncta size, nuclear LC3B pixel intensity, perinuclear LC3B puncta per cell and perinuclear LC3B puncta size in control and BafA1-treated WT and *Nesprin 2*^*-/-*^ E14.5 MEFs from Fig. 2e. The box plots represent the 25th–75th percentiles, the lines depict the median and the whiskers show the min–max values. ****P<0.0001 using two-way ANOVA; n ≥ 44 puncta, n ≥ 31 nuclei, n ≥ 20 cells and n ≥ 8 puncta respectively. Source data

We next asked whether nesprin protein family members function as regulators of autophagy-related events, such as omegasome formation, autophagosome formation and autophagic protein degradation. Knockdown of the *anc-1* gene, encoding the nematode nesprin ortholog, causes accumulation of double FYVE-containing protein 1 (DFCP1)::GFP puncta, signifying early autophagic structures^19^ in somatic tissues of adult *C. elegans* animals (Fig. 2a,b). Notably, depletion of ANC-1 under autophagy-related protein 2 (ATG-2) deficiency, a protein required for pre-autophagosomal structure organization, does not increase the number of DFCP1::GFP puncta further, indicating that both ANC-1 and ATG-2 function in the same pathway (Fig. 2a,b). We observed a similar elevation of LGG-1 puncta formation on ANC-1 depletion (Fig. 2a,b). We then assessed the requirement of ANC-1 under low insulin/insulin-like growth factor I (IGF-1) signaling, known to induce autophagy in *daf-2* insulin/IGF-1 receptor mutant nematodes^20–22^. ANC-1 deficiency makes autophagosomal puncta larger, indicating that autophagosomes either increase in size or coalesce (Fig. 2a,b). Absence of ANC-1 induces accumulation of sequestosome related-1 (SQST-1), the *C. elegans* ortholog of p62 (Fig.2a,b). Genetic inhibition of early Beclin homolog 1 (*bec-1*) and late (*rab-7*) stage autophagy indicate that ANC-1 regulates late autophagic steps, since *bec-1* downregulation showed further increase in GFP::LGG-1 accumulation compared to animals with *anc-1* downregulation (Extended Data Fig. 3a,b). In addition, *rab-7* downregulation failed to further accumulate GFP::LGG-1 compared to animals with *anc-1* downregulation. Collectively, these observations indicate that ANC-1 is implicated in late autophagic steps; therefore, its absence affects progression and/or completion of the autophagic process. We used a polyglutamine (Q40) disease model, where polyglutamine aggregates form^23^. Autophagy has been shown to degrade such aggregate-prone proteins with polyglutamine expansion^24,25^. Remarkably, we show that young adult nematodes accumulate more puncta in the absence of ANC-1 (Fig. 2c,d). A similar phenotype was observed when ANC-1 and LGG-1 or BEC-1 were silenced in parallel, implying that ANC-1 suppresses the accumulation of polyglutamine aggregates through autophagy (Fig. 2c,d).

We then sought to corroborate the unexpected function of ANC-1 as an autophagy regulator in the mouse. To this end, we assessed the levels and distribution of LC3B and p62. Consistent with this notion, LC3B puncta were more abundant in *nesprin-2*^−/−^ MEFs (Fig. 2e,f). Autophagic flux inhibition (BafA1) did not further increase LC3B puncta accumulation in *nesprin-2*^−/−^ MEFs. In conjunction, in the absence of nesprin-2, the LC3II/I ratio exhibited an increased trend, albeit non-significant, indicating possibly defective autophagic clearance (Fig. 2g,h). In addition, LC3 puncta size was larger under autophagic flux inhibition, indicating autophagosome aggregation, when nesprin-2 was depleted (Fig. 2e and Extended Data Fig. 3c). p62 accumulated in the nucleus under basal conditions in the absence of nesprin-2 (Fig. 2i,j). Notably, we observed an aggregation of nuclear p62 (Fig. 2i,j), coupled with a nuclear and perinuclear accumulation of LC3B in the absence of nesprin-2 (Fig. 2e and Extended Data Fig. 3d–f), further suggesting a role of nesprin-2 in autophagy. Combined, these findings uncover an evolutionarily conserved role for nesprin protein family members in autophagy that could influence and, possibly, coordinate organelle-specific homeostasis, including the nucleus.

### Autophagy and ANC-1/nesprin-2 control nucleolar homeostasis

**Fig. 3:**
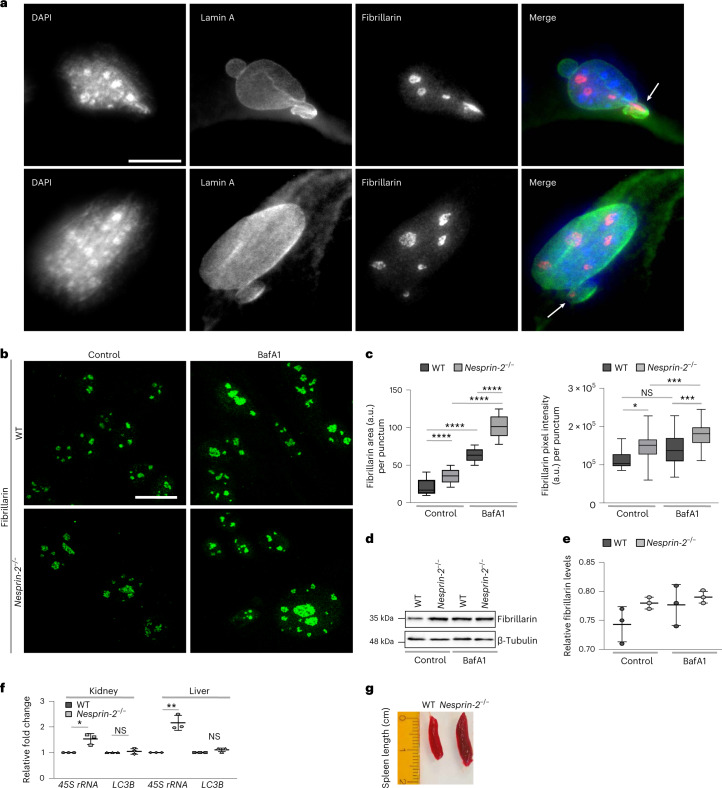
Autophagy and nesprins regulate nucleolar components and ribosomal biogenesis. **a**, Epifluorescence microscopy imaging of WT E14.5 MEFs stained with DAPI (blue) and immunostained for endogenous lamin A (green) and endogenous fibrillarin (red). The arrows indicate nuclear protrusions positive for fibrillarin. Scale bar, 20 μm. **b**, Confocal microscopy imaging of WT and *nesprin-2*^−/−^ E14.5 MEFs, under control or BafA1 treatment, immunostained for endogenous fibrillarin. Scale bar, 20 μm. **c**, Quantification of fibrillarin area per punctum and pixel intensity per punctum (indicative of the nucleolus) from **b**. The box plot represents the 25th–75th percentiles, the line depicts the median and the whiskers show the min–max values. NS ≥ 0.05, *P < 0.05, ***P < 0.001, ****P < 0.0001 using a one-way ANOVA; *n* ≥ 22 puncta. **d**, Western blot analysis of fibrillarin in WT and *nesprin-2*^−/−^ E14.5 MEFs under control or BafA1 treatment. **e**, Quantification of fibrillarin protein levels normalized to β-tubulin levels from **d**. Mean ± s.d. of three independent experiments. **f**, Quantification of kidney and liver relative *45S rRNA* and *LC3B* transcript levels by qPCR of 6-month-old WT and *nesprin-2*^−/−^ mice, normalized to *HMBS* transcript levels. Mean ± s.d. of three independent experiments. NS ≥ 0.05, *P < 0.05, **P < 0.01 using an unpaired *t*-test. **g**, Spleens dissected from 6-month-old WT and *nesprin-2*^−/−^ male mice. The ruler indicates length. Source data

Previous studies in diverse organisms, ranging from nematodes to humans, indicate that nucleolar size is a determinant of longevity under conditions of low insulin/IGF-1 signaling and dietary restriction^9,26–28^. However, the mechanisms responsible for nucleolar size regulation are elusive. Given the requirement of nesprins for autophagy under conditions of low insulin/IGF-1 signaling and nutrient deprivation, we investigated whether nesprin-mediated autophagy impinges on the nucleolus. Fibrillarin is a predominantly nucleolar protein, implicated in nucleolar size regulation^7^. Similarly to nesprin-2 and lamin A, it is found in nuclear bulges that have been implicated in nucleophagic degradation (Fig. 3a). Remarkably, we found that fibrillarin is a substrate of autophagy (Fig. 3b,c). Of note, depletion of nesprin-2 or blockage of basal autophagic flux in MEFs causes accumulation of fibrillarin and fibrillarin puncta expansion (Fig. 3b,c). We corroborated these findings by western blot analysis in nesprin-2-depleted MEFs where fibrillarin levels were increased on autophagic flux inhibition, albeit not significantly due to possible functional redundancy of nesprin-1 and 2 (Fig. 3d,e). Thus, nesprin-2 controls the amount of nucleolar fibrillarin via autophagy. In addition to fibrillarin, nesprin-2 modulates the expression of the 45S ribosomal RNA that is required for proper and efficient oocyte development, without interfering with the expression of core autophagy genes, such as *LC3B* (Fig. 3f). Lastly, we found that 5-month old, *nesprin-2*^−/−^ mice displayed splenomegaly (Fig. 3g), which is a pathological condition related to lysosomal storage diseases^29^. These observations suggest that nesprins affect nucleolar size and function, likely through autophagic degradation of nuclear and nucleolar material.

**Fig. 4:**
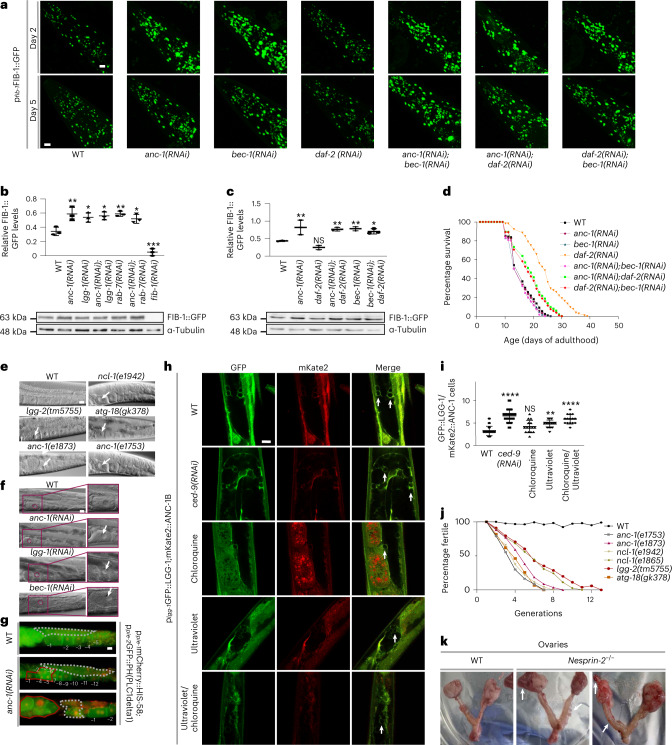
Nucleophagy promotes germline immortality and longevity of the soma by restricting nucleolar expansion. **a**, Confocal microscopy imaging of day 2 and day 5 adult worms expressing p_*fib-1*_FIB-1::GFP, subjected to RNAi-mediated silencing of the indicated genes. Scale bar, 20 μm. **b**, Western blot analysis and quantification of relative FIB-1::GFP protein level expression of day 3 adult worms subjected to RNAi-mediated silencing of the *anc-1*, *lgg-1*, *anc-1*;*lgg-1*, *rab-7*, *anc-1*;*rab-7* and *fib-1* genes. Mean ± s.d. of three biological replicates. *P < 0.05, **P < 0.01, ***P < 0.001 using a one-way ANOVA. **c**, Western blot analysis and quantification of relative FIB-1::GFP protein level expression of day 3 adult worms subjected to RNAi-mediated silencing of the *anc-1*, *daf-2*, *anc-1*;*daf-2*, *bec-1* and *anc-1*;*bec-1* genes. Mean ± s.d. of three biological replicates. NS ≥ 0.05, *P <0.05, **P<0.01 using a one-way ANOVA. **d**, Life span analysis of RNAi-mediated silencing of *anc-1* or *bec-1* in a WT and DAF-2-deficient background; *n* ≥ 131 worms. **e**, DIC microscopy imaging of day 2 worm gonads subjected to RNAi-mediated silencing of the indicated genes at 25 °C. Arrows indicate the most proximal nucleoli. Scale bar represents 20μm. **f**, DIC microscopy imaging of day 2 worm gonads subjected to RNAi-mediated silencing of with *anc-1*, *lgg-1* and *bec-1* at 25 °C. The zoomed areas indicate each most proximal oocyte. The arrows indicate the nucleoli of the most proximal oocyte. Scale bar, 20 μm. **g**, Epifluorescence microscopy imaging of WT and *anc-1(RNAi)*-silenced worms expressing p_*pie-1*_mCherry::HIS-58;p_*pie-2:*_GFP::PH(PLC1delta1), indicating nuclear (red) and cell membrane (green) boundaries respectively at 25 °C. The numbers indicate oocytes starting from the most proximal (−1) to the most distal oocyte. The gray dashed lines highlight the germ cell area. The red lines highlight tumor-like structure (middle image) or tumor formed (lower image). Scale bar, 20 μm. **h**, Confocal microscopy imaging of day 2 adult worms expressing p_*lgg-1*_GFP::LGG-1 and mKate2::ANC-1B, subjected to RNAi-mediated silencing of the *ced-9* gene, treated with chloroquine or ultraviolet/chloroquine at 25 °C. The arrows indicate GFP/mKate double-positive cells. Scale bar, 10 μm. **i**, Quantification of double GFP- and mKate2-labeled germ cells. Mean ± s.d. NS ≥ 0.05, **P < 0.01, ****P < 0.0001 using a one-way ANOVA; *n* = 15 worm midbody areas. **j**, Percentage of fertility of indicated worm strains across generations. **k**, Dissected ovaries from 1.5-year-old mice. The arrows indicate ovarian tumors in *nesprin-2*^−/−^ female mice. Source data

**Extended Data Fig. 4:**
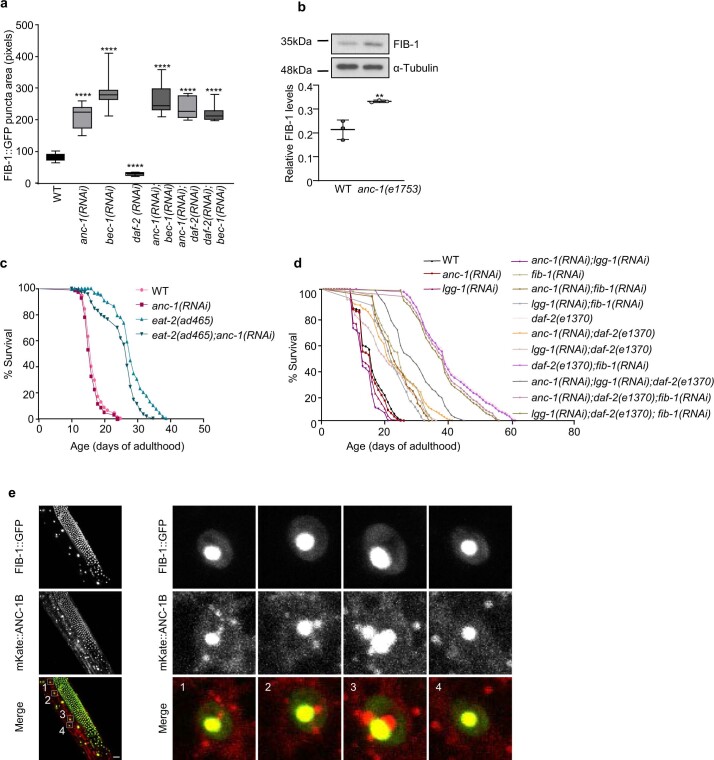
ANC-1 modulates nucleolar size. **a**, Quantification of FIB-1::GFP puncta area from Fig. 4a. The box plot represents the 25th–75th percentiles, the line depicts the median and the whiskers show the min–max values. ****P<0.0001 using one-way ANOVA; n ≥ 24 puncta. **b**, Western blot analysis and quantification of relative endogenous FIB-1 levels of day 3 adult WT worms and *anc-1(e1753)* mutants. Mean ± s.d. of three biological replicates. **P<0.01 using unpaired *t*-test. **c**, Life span analysis of EAT-2-deficient worms subjected to RNAi-mediated silencing of *anc-1*. **d**, Life span analysis of long-lived DAF-2- and FIB-1-deficient worms subjected to RNAi-mediated silencing of *anc-1* and *lgg-1*. n ≥ 88 worms **e**, Confocal microscopy imaging of FIB-1::GFP;mKate::ANC-1-expressing day 2 worms. Enlarged images of the indicated numbered squares are shown on the right panel. Scale bar, 20μm. Source data

To investigate the physiological significance of nesprin-2-mediated effects on the nucleolus in vivo, we monitored nucleolar structure perturbations in *C. elegans*, using a fibrillarin reporter strain (FIB-1::GFP). Notably, ANC-1 deficiency caused enlargement of nucleoli and increased endogenous FIB-1 protein levels, in otherwise WT animals, which is recapitulated by depletion of the *C. elegans* Beclin ortholog BEC-1 (Fig. 4a–c and Extended Data Fig. 4a,b). No further increase was observed by simultaneous knockdown of both ANC-1 and BEC-1, suggesting that they function in the same pathway to influence nucleolar size, while autophagic perturbation by either *lgg-1* or *rab-7* silencing alone or with simultaneous *anc-1* silencing also increases FIB-1 protein levels (Fig. 4b,c and Extended Data Fig. 4a). By contrast, autophagy-inducing, low-insulin/IGF-1 signaling (on *daf-2* knockdown) results in reduced nucleolar size and FIB-1 protein levels (Fig. 4a,c and Extended Data Fig. 4a). Importantly, either *anc-1* or *bec-1* knockdown is epistatic to DAF-2 deficiency (Fig. 4a,c,d). These observations indicate that ANC-1 and autophagy contribute downstream of insulin/IGF-1 signaling to preserve nucleolar morphology and homeostasis in *C. elegans*.

### ANC-1 regulates somatic aging

Given the requirement of nesprin protein family members for autophagic recycling of nucleolar components such as fibrillarin, under both basal and nutrient stress conditions in diverse organisms, we examined whether nesprins also mediate the pro-longevity effects of low insulin/IGF-1 signaling and dietary restriction. Remarkably, knockdown of *anc-1* shortened the life span of long-lived DAF-2-deficient nematodes, to an extent similar to that of BEC-1 depletion (Fig. 4d). Hence, ANC-1 functions to maintain nucleolar homeostasis and promote longevity under low insulin/IGF-1 signaling conditions. Similarly, loss of ANC-1 shortens the life span of long-lived caloric-restricted neuronal acetylcholine receptor subunit (EAT-2)-deficient animals (Extended Data Fig. 4c). FIB-1 silencing has been shown to increase life span; however, neither ANC-1 nor LGG-1 ablation further altered the life span, indicating that they act upstream of FIB-1 (Extended Data Fig. 4d). Moreover, *fib-1(RNAi)* does not increase the life span of long-lived *daf-2* mutants (Extended Data Fig. 4d).

### ANC-1 and autophagy promote gonad integrity and germline immortality

**Fig. 5:**
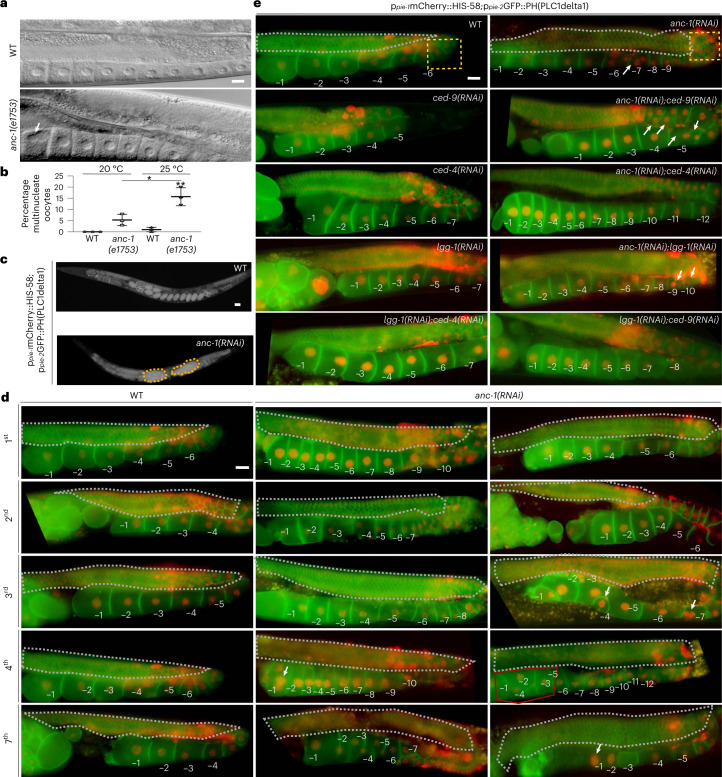
ANC-1 preserves germline anatomy and acts as a tumor suppressor. **a**, DIC microscopy imaging of day 2 WT and *anc-1(e1753)* mutant worm gonads. Scale bar, 20 μm. **b**, Quantification of percentage of multinucleate oocytes from **a**. Mean ± s.d. of three biological replicates. *P < 0.05, **P < 0.01 using a two-way ANOVA. **c**, Epifluorescence microscopy imaging of WT and *anc-1(RNAi)*-treated p_*pie-1*_mCherry::HIS-58;p_*pie-2*_GFP::PH(PLC1delta1)-expressing worms after 5 generations at 25 °C. Scale bar, 20 μm. **d**, Epifluorescence microscopy imaging of gonads of p_*pie-1*_mCherry::HIS-58;p_*pie-2*_GFP::PH(PLC1delta1)-expressing WT and *anc-1(RNAi)*-mediated silenced worms grown at 25 °C after 1, 2, 3, 4 and 7 generations. The numbers indicate oocytes starting from the most proximal (−1) to the most distal. The gray dashed lines highlight germ cell areas. The arrows indicate multinuclearity or aberrant oocytes. The red line indicates a tumor-like structure. Scale bar, 20 μm. **e**, Epifluorescence microscopy imaging of gonads of p_*pie-1*_mCherry::HIS-58;p_*pie-2*_GFP::PH(PLC1delta1)-expressing worms after RNAi-mediated silencing of the indicated genes. The numbers indicate oocytes starting from the most proximal (−1) to the most distal. The gray dashed lines highlight the germ cell area. The yellow dashed squares highlight gonad turn where apoptotic cell death occurs. The arrows illustrate multinucleate oocytes. Scale bar, 20 μm. Source data

**Extended Data Fig. 5:**
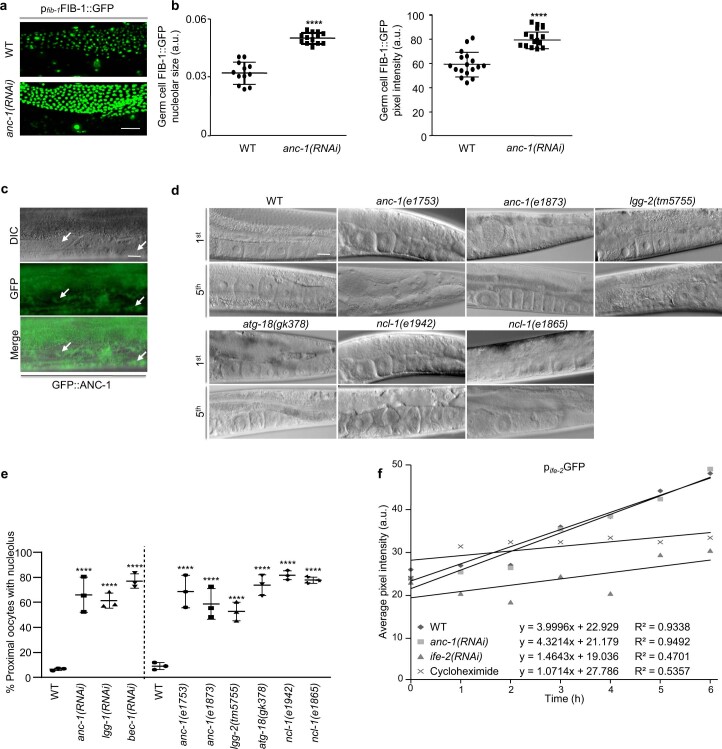
ANC-1 preserves germline anatomy. **a**, Confocal microscopy imaging of day 2 WT and *anc-1(RNAi)*-mediated silenced FIB-1::GFP-expressing worm gonads grown at 25 °C. Scale bar, 10μm. **b**, Quantification of germ cell FIB-1::GFP nucleolar size and pixel intensity in WT and *anc-1(RNAi)*-mediated silenced worms from **a**. Mean ± s.d. ****P<0.0001 using one-way ANOVA; n ≥ 12 nucleoli. **c**, Epifluorescence microscopy imaging of GFP::ANC-1-expressing worm gonads. Arrows indicate distinct nucleoli visible by DIC microscopy, which are encircled by GFP::ANC-1. Scale bar, 10μm. **d**, DIC microscopy imaging of day 2 gonads of WT worms and gonads of worms subjected to RNAi-mediated silencing of the indicated genes grown at 25 °C after one generation and five generations. Scale bar, 20μm. **e**, Percentage of worm gonads grown at 25 °C whose proximal oocyte contains a nucleolus. Mean ± s.d. of three biological replicates. ****P<0.0001 using one-way ANOVA. **f**, Monitoring of protein synthesis rates by fluorescence recovery after photobleaching (FRAP) analysis. FRAP was performed on day 1 WT control, *anc-1(RNAi)-*, *ife-2(RNAi)-* or cycloheximide-treated (protein synthesis inhibitor) worms, expressing the reporter p_*ife-2*_GFP. The *ife-2* gene encodes the predominant eIF4E isoform in *C. elegans* that upon depletion blocks protein synthesis. The best-fit line illustrates the recovery rate, while the slope is the quantification of the recovery rate of a representative experiment. Source data

Strikingly, we observed distinct differences in proximal oocyte nucleolar size in the reproductive system of the nematode. Physiologically, small-sized proliferating germ cells in the gonad contain big nucleoli, which, as they enter meiosis and differentiate into oocytes, decrease their nucleolar size^27^. The most proximal oocyte, which will be fertilized, has no nucleolus. This process of regulating nucleolar size in the gonad is integral to germ cell differentiation and healthy oocyte production. To dissect this process, we examined ANC-1 expression in the gonad. ANC-1 colocalizes with fibrillarin in nucleoli (Extended Data Fig. 4e), while in the absence of ANC-1, proliferating gonadal germ cells have larger nucleoli (Extended Data Fig. 5a–c). Proximal oocytes fail to eliminate their nucleoli on ANC-1 or autophagic protein knockdown (Fig. 4e,f and Extended Data Fig. 5d,e). Autophagy controls nucleolar elimination at the proximal oocyte, as autophagy-deficient animals (*lgg-2(tm5755)*, *atg-18(gk378)*, *lgg-1(RNAi)* and *bec-1(RNAi)*), similar to the FIB-1 transcriptional repressor nucleolin (*ncl-1*) mutants, have prominent nucleoli at their proximal oocytes (Fig. 4e,f and Extended Data Fig. 5d,e) without impacting the rate of protein synthesis in vivo (Extended Data Fig. 5f)^30^. Worms grown at 25 °C lacking B-box type zinc finger protein NCL-1, autophagic components or ANC-1, display severe anatomical gonadal abnormalities after five generations and form tumor-like structures (Extended Data Fig. 5d and Fig. 4g). LGG-1 and ANC-1 colocalize in gonadal germ cells, indicating their functional interaction in the gonad (Fig. 4h,i). Notably, ANC-1-, autophagy- or NCL-1-deficient nematodes, which cannot shed their nucleoli in parallel to gonadal collapse, show progressive decline in fecundity under stress, with diminished egg laying capacity after five generations at 25 °C (Fig. 4j). Thus, nucleoli persistence at the most proximal oocytes seem to have physiological implications concerning germline immortality and act as a biomarker of gradual reduction in egg laying capacity. Apart from tumor formation, oocyte multinuclearity was also observed, similar to mammalian malignancies (Fig. 5a–c). Similar to tumors formed in the reproductive system of ANC-1-deficient hermaphrodite nematodes, female mice lacking nesprin-2, exhibit ovarian and endometrial tumors, showing evolutionary functional conservation (Fig. 4k)^31^. Analysis of ANC-1-depleted gonads across generations reveals gradual gonadal deterioration (Fig. 5d). Specifically, we observe degenerating features such as smaller aberrant oocytes (second generation), which later misalign with each other (third generation). Moreover, gonads exhibit malignancy-like phenotypes such as multinuclearity (third and fourth generation), tumor-like structures (fourth generation) and loss of cellular integrity (seventh generation) (Fig. 5d).

**Fig. 6:**
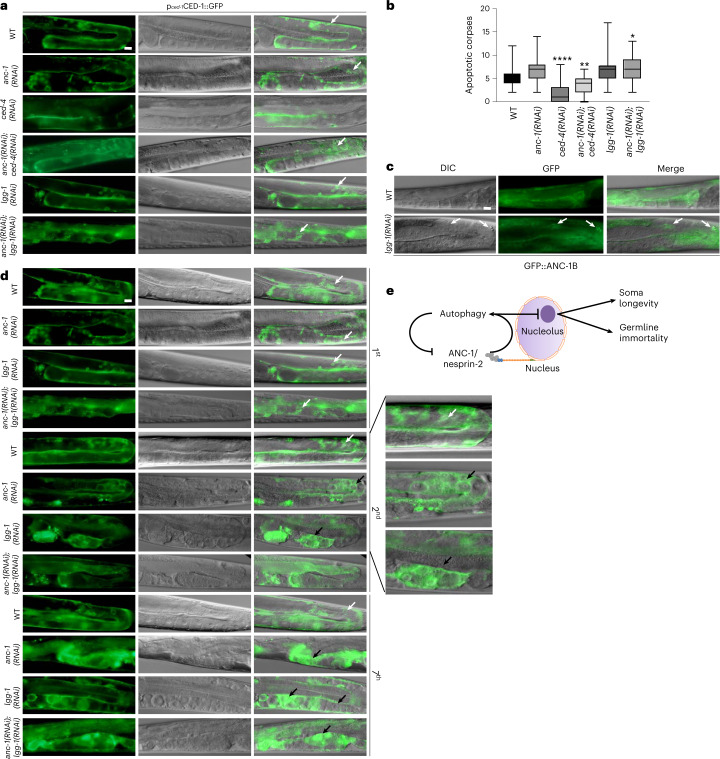
ANC-1 modulates germ cell death. **a**, Epifluorescence and DIC microscopy imaging of p_*ced-1*_CED-1::GFP-expressing worms subjected to RNAi-mediated silencing of the indicated genes. The arrows indicate apoptotic corpses. Scale bar, 20 μm. **b**, Quantification of apoptotic corpses using p_*ced-1*_CED-1::GFP-expressing worms from **a**. The box plot represents the 25th–75th percentiles, the line depicts the median and the whiskers show the min–max values. *P < 0.05, **P < 0.01, ****P < 0.0001 using one-way ANOVA; *n* ≥ 42 worm gonads. **c**, Epifluorescence and DIC microscopy imaging of WT and *lgg-1(RNAi)-*treated GFP::ANC-1-expressing worm gonads, illustrating ANC-1 expression in somatic gonadal sheath cells and apoptotic corpses. The arrows indicate apoptotic corpses encircled by GFP::ANC-1. Scale bar, 20 μm. **d**, Epifluorescence and DIC microscopy imaging of p_*ced-1*_CED-1::GFP-expressing worms subjected to RNAi-mediated silencing of *anc-1* and *lgg-1* genes after 1, 2 or 7 generations grown at 25 °C. The white arrows indicate apoptotic corpses. The black arrows indicate abnormally enlarged CED-1-decorated structures. The magnification of second-generation gonads is shown. Scale bar, 20 μm. **e**, Schematic representation of the cellular pathway by which autophagic recycling of nuclear envelope-associated and nucleolar components preserves germline immortality and delays aging under conditions of stress. Source data

This quality control mechanism involving ANC-1 and LGG-1 is additive and synergistic with the core apoptotic cell death machinery. ANC-1 germ cell quality assurance can potentially act through a recently described autophagic cell death engulfment mechanism involving LGG-1, which has been shown to act cooperatively with the pro-apoptotic cell death abnormality protein 1 (CED-1) to degrade cells with accumulated damage (Fig. 5e and Fig. 6a–b)^32^. Corroborating ANC-1’s involvement in the clearance of dying cells, it colocalizes with LGG-1 under apoptosis-inducing conditions, either by silencing of prosurvival apoptosis regulator Bcl-2 ortholog CED-9 or by inducing DNA damage (ultraviolet) (Fig. 4h,i). Moreover, absence of LGG-1, ANC-1 or both causes accumulation of oocytes (Fig. 5e). We observed that on *lgg-1* silencing, ANC-1 is localized to apoptotic corpses, either aggregating or forming a circular structure around the apoptotic corpses, which is reminiscent of the CED-1 pattern (Fig. 6c). Importantly, the gonads of worms grown at 25 °C for several generations show increased cellular damage in the absence of ANC-1, LGG-1, or both. They display gonads with abnormally large CED-1-decorated structures, which are indicative of aggregated germ cells/apoptotic corpses that cannot be cleared out due to their defective autophagic engulfment (Fig. 6d). These finding indicate that, similarly to DNA damage repair and chromosomal maintenance^33,34^, autophagic clearance of superfluous/damaged germ cells in the gonad may contribute to sustain germline immortality.

## Discussion

Our study reveals that autophagic recycling of nuclear material is a key cellular process that preserves nuclear architecture, restricts nucleolar size and promotes longevity. Nuclear envelope nesprin proteins may function as specific mediators of autophagic degradation of nuclear components. Nesprin-2 impairment triggers nuclear protein aggregation and accumulation of enlarged autophagosomal structures in the cytoplasm. Notably, nesprins are required to maintain small nucleoli, a distinctive feature of long-lived genetic backgrounds, and life span-extending regimes. In addition to identifying ANC-1/nesprin-2 as a downstream effector of low insulin/IGF-1 signaling and dietary restriction on somatic aging, we also found that autophagic recycling of nuclear and nucleolar components is a key contributor to germline immortality, through autophagic clearance of germ cell corpses. Indeed, ANC-1 lesions cause germline mortality and progressive sterility (the Mortal Germline phenotype)^35^ in *C. elegans*, while *nesprin-2* polymorphisms have been associated with infertility, endometriosis and ovarian cancer in humans^36^. Thus, ANC-1/nesprin-2 promotes an important longevity assurance mechanism that upholds germline immortality and contributes downstream of common life-prolonging interventions to maintain nuclear homeostasis and halt nucleolar expansion (Fig. 6e). Our findings provide a molecular basis for bridging the dichotomy between two diametrically opposed, fundamental phenomena: somatic aging and germline immortality. Nuclear recycling upregulation in the disposable soma may endow it with germline-like properties, thus extending somatic life span^34,37,38^. The tight evolutionary conservation and ubiquitous expression of nodal autophagy regulators suggest that similar pathways promote youthfulness and delay aging across distant taxa.

## Methods

### *C. elegans* strains

Strains were maintained at 20 °C unless otherwise noted. All experiments were carried out using hermaphrodite worms. The following strains were obtained from the *Caenorhabditis* Genetics Center (https://cgc.umn.edu/): N2: WT Bristol isolate; AM141: *rmIs133* (*unc-54p::Q40::YFP*); CB1370: *daf-2(e1370)*; CB3335: *anc-1(e1802)*; CB3339: *anc-1(e1753)*; CB3388: *ncl-1(e1865)*; CB3440: *anc-1(e1873)*; CF2218: *ncl-1(e1942)*; COP262: *knuSi221* (*fib-1p::fib-1(genomic)::eGFP::fib-1 3’ UTR* + *unc-119(+)*); CU1546: *smIs34* (*ced-1p::ced-1::GFP* + *rol-6(su1006)*); DA2123: *adIs2122* (*lgg-1p::GFP::lgg-1* + *rol-6(su1006)*); HZ589: *him-5(e1490)*;*bpIs151* (*sqst-1p::sqst-1::GFP* + *unc-76(+)*); LW697: *ccIs4810* (*(pJKL380.4) lmn-1p::lmn-1::GFP::lmn-1 3’utr* + *(pMH86) dpy-20(+)*); MAH14: *daf-2(e1370)*;*adIs2122* (*lgg-1::GFP* + *rol-6(su1006)*); OD95: *unc-119(ed3)*;*ltIs37* (*pie-1p::mCherry::his-58* + *unc-119(+)*);*ltIs38* (*pie-1p::GFP::PH(PLC1delta1)* + *unc-119(+)*); and VC893: *atg-18(gk378)*. *lgg-2(tm5755)* was obtained from the National Bioresource Project (https://shigen.nig.ac.jp/c.elegans/top.xhtml). DA465: *eat-2(ad465)* was a kind gift from L. Avery. *bpIs168* (*nfya-1p::dfcp-1::GFP; unc-76(+)*) and *bpIs168* (*nfya-1p::dfcp-1::GFP; unc-76(+)*);*atg-2(bp576)* were kind gifts from H. Zhang. UD612: *yc68* (*gfp::anc-1b*) and UD626: *yc72* (*mKate2::anc-1b*) were kind gifts from D. Starr. IR593 N2;*Ex*(*ife-2p::ife-2::GFP* + *rol-6(su1006)*), IR2969: *yc72* (*mKate2::anc-1b*);*adIs2122* (*lgg-1p::GFP::lgg-1* + *rol-6(su1006)*) and IR2970: *yc72* (*mKate2::anc-1b*);*knuSi221* (*fib-1p::fib-1(genomic)::eGFP::fib-1 3*′-*UTR* + *unc-119(+)*) were generated in this study.

### Molecular cloning

RNAi constructs were engineered by PCR amplification of genomic DNA using the following primer sets: *anc-1*: forward: 5′-AAGAGTTGAGACGTGCTCTCC-3′, reverse: 5′-ACAGGATCATCGATTGTGTCCAG-3′; *bec-1*: forward: 5′-GCTCTAGAGTTATCACAGAAGCTCTG-3′, reverse: 5′-GCTCTAGAGTTATCACAGAAGCTCTG-3′; *daf-2*: forward: 5′-CGGGATCCTGTGCCCACGTGGAGCTT-3′, reverse: 5′-CCGCTCGAGTGAATAGCGTCCGAATCGA-3′; *lgg-1*: forward: 5′-GGAATTCAAGTGGGCTTACAAGGAG-3′, reverse: 5′-GGAATTCGTCTTCTTCGTTTATTCATG-3′; *rab-7*: forward: 5′-TTGTCGTTCAATTTCGGTGA-3′, reverse: 5′-TGAAAACGGCTTGGAAGTTT-3′; *ced-*4: forward: 5′-AAGCTTTGCTGAATGAGCGATTACGAC-3′, reverse: 5′-GGATCCAGCTCGGCCGTGTAGAAACAG-3′; *ced-9*: forward: 5′-ATGACACGCTGCACGGCGG-3′, reverse: 5′-CTTCAAGCTGAACATCATCCGCCC-3′; *fib-1*: forward: 5′-CTCATACGGCTCCAGGGTTA-3′, reverse: 5′-TGTTCGCCATCAAAACGATA-3′; *ife-2*: forward: 5′-CGGGATCCAGCAAGTAATGTCCGAAG-3′, reverse: 5′-AACTGCAGCATTTCAACAAGTGAAGAAC-3′.

The resulting fragments were subcloned into the pL4440 plasmid vector. Each plasmid construct was then transformed into HT115(DE3) *Escherichia coli* bacteria. Bacteria carrying an empty plasmid vector were used as control.

### Stress assays

Worms were synchronized by treating gravid adults with bleaching solution (H_2_O:bleach:5 N NaOH 7:2:1). Animals were washed three times in M9 buffer and incubated in M9 buffer on a rotor. On days 5 and 10, 30 μl of M9 were plated on an NGM plate; after 1 h, the percentage of alive larval stage 1 worms was measured. For the colocalization assays, starvation was performed for 6 h and chloroquine (catalog no. 1825; BioVision) was added on plates at 5 μM concentration 3 h before imaging. For heat stress, day 1 adult animals were placed in a 37 °C incubator for 1 h and survival was scored on days 2 and 4 of adulthood. For ultraviolet-C-induced DNA damage, day 1 worms were placed on ultraviolet-inactivated OP50 bacteria and treated with either 200 J m^−^^2^ or 400 J m^−^^2^ ultraviolet-C irradiation using an ultraviolet crosslinker (BIO-LINK-BLX-E365; Vilber Lourmat).

### Egg laying assay and gonad microscopy

Larval stage 4 worms from each strain (10 worms per strain) were individually placed on NGM plates at 20 °C and 25 °C. Eggs were counted and removed for the next 3 d. Ten larvae of the next generation (offspring) were transferred from each plate of each strain to measure the egg laying capacity. This was repeated until no eggs were detected in the plates of *anc-1* mutant worms placed at 25 °C. Fluorescence and differential interference contrast (DIC) microscopy of gonadal structure were performed at 25 °C unless otherwise stated since it is considered a mild heat stress and is the relevant temperature to test germline immortality.

### Fluorescence recovery after photobleaching assays

Day 1 adult worms were treated with RNAi for 24 h or the protein translation inhibitor cycloheximide (500 μg ml^−1^) 2 h before photobleaching. Photobleaching was performed using an epifluorescence microscope for 7 min (Axioskop 2 Plus; ZEISS). Images were captured before, immediately after and every hour for the consecutive 6 h after photobleaching.

### Life span assays

Life span assays under RNAi were performed at 20 °C unless otherwise noted. Synchronization of animal populations was performed by hypochlorite treatment of gravid adults on RNAi plates containing 2 mM isopropyl β-d-1-thiogalactopyranoside and seeded with HT115(DE3) *E. coli* bacteria, transformed with the pL4440 plasmid vector. Next-generation, larval stage 4 animal populations were then reared on NGM plates, seeded with HT115(DE3) *E. coli* bacteria, transformed with either the pL4440 plasmid vector or the specific RNAi plasmid construct. Twenty animals were used per plate for a total of 150–200 individuals per experiment. Animals were transferred to fresh plates every 2–4 d. Touch-provoked movement and pharyngeal pumping were assessed until death. Animals that died due to gonad extrusion or internal egg hatching were censored. Survival curves were repeated at least twice and representative graphs were used in the figures. Survival curves were created using the product-limit method of Kaplan and Meier. The log-rank (Mantel–Cox) test was used to evaluate differences between survivals and determine *P* values. We used the Prism software (GraphPad Prism 8.0) for statistical analysis and to determine life span values.

### Imaging and quantification

Imaging was performed with a ZEISS AxioImager Z2 Epifluorescence or a ZEISS LSM 710 confocal microscope or EVOS FL Auto 2 imaging system (AMAFD2000; Thermo Fisher Scientific). Specifically, LMN-1-expressing cells were scored for nuclear irregularity according to the existence of (1) micronuclei, (2) LMN-1 aggregation and (3) irregular shape, such as nuclear envelope invagination or folding. The percentage of irregular nuclei was measured as an output of the number of irregular nuclei per worm to the total number of cells in the image. Measurement of LMN-1::GFP pixel intensity in the *C. elegans* head and intestinal cells was performed by quantifying the maximum pixel intensity of the Z-stacks of individual GFP-expressing cells. For GFP::LGG-1 pixel intensity quantification in the head, maximum projection images were acquired using the super-apochromat Olympus 20× objective (AMEP4734) of the EVOS FL Auto 2 imaging system with identical settings for all conditions. The background fluorescence signal was subtracted, head regions were segmented and pixel intensity was measured in ImageJ 1.53f51 bundled with 64-bit Java 1.8.0_66. For the number of GFP::LGG-1 puncta, GFP puncta were counted at the head region of each worm from confocal images using the maximum pixel intensity of the Z-stacks. In *daf-2(e1370);*p_*lgg*-*1*_GFP::LGG-1, the GFP puncta area was measured using the maximum pixel intensity of the Z-stacks. In all quantifications, background subtraction and threshold setting were performed.

### Mouse models

All animal protocols were approved by the FORTH Ethics Committee and the Directorate of Agricultural and Veterinary Political Region of Crete (MacroAutophagy and Necrotic Neurodegeneration in Ageing, protocol no. 7853). All mice were maintained in a pathogen-free environment and housed in clear shoebox cages, in groups of 5 animals per cage with constant temperature and humidity and a 12 h light–12 h dark cycle. Apart from the ovarian anatomical studies, all mice used where males of C57BL/6 or *nesprin-2*^−/−^ genetic background. Adult animals were used as indicated in the text. Food deprivation was initiated in the morning, for a total duration of 24 h, with free access to water.

### Quantitative PCR with reverse transcription

For messenger RNA quantification, total RNA isolation from MEFs, liver and kidneys was performed using the TRIzol reagent (Thermo Fisher Scientific). Complementary DNA synthesis was performed using the iScript kit (Bio-Rad Laboratories). Quantitative PCR (qPCR) was performed with the Eva Green qPCR Kit (Biotium). All kits were used according to the manufacturer’s instructions. The following oligonucleotides were used for mRNA quantification: *45S* *rRNA*: forward:5′-GATGTGTGAGGCGCCCGGTT-3′ and reverse: 5′-GTATGCAACGCCACCGGCCA-3′; *LC3b*: forward: 5′-CGTCCTGGACAAGACCAAGT-3′ and reverse: 5′-ATTGCTGTCCCGAATGTCTC-3′; *nesprin-2*: forward: 5′-CGAGCTGGAAGCTCTGAAGT-3′ and reverse: 5′-ATGGAGTCTATTTTGGAGTTCTGTG-3’; and hydroxymethylbilane synthase (*HMBS*): forward: 5′-GATGGGCAACTGTACCTGACTG-3′ and reverse: 5′-CTGGGCTCCTCTTGGAATG-3′.

### Immunoblotting and immunoprecipitation

Mouse liver was isolated and immediately processed for protein analysis by western blot. Briefly, tissues were collected in ice-cold PBS and lysed by sonication in radioimmunoprecipitation assay (RIPA) buffer (50 mM Tris-HCl, pH 7.8, 150 mM NaCl, 0.5% sodium deoxycholate, 0.1% SDS, 1% Triton X-100), supplemented with protease and phosphatase inhibitors (Roche), placed for 20 min on ice, followed by a 20 min centrifugation at 14,000*g*. For coimmunoprecipitation, overnight preclearing of the protein samples was performed with 30 μl protein G agarose beads and placed on a moving rotor at 4 °C. The next day, the protein lysate was transferred to a clean tube, with fresh beads, and the antibody of interest or control IgG antibody of the same species incubated overnight on a moving rotor at 4 °C. The following day, three washes with RIPA were performed followed by Laemmli addition, 10 min boiling at 95 °C followed by transfer of the supernatant without beads to a clean tube. *C. elegans* proteins were extracted from at least 100 worms per strain per condition using RIPA and 6× Laemmli buffer with 5 min vortexing followed by 10 min incubation at 95 °C. Samples were run on 10–15% polyacrylamide gels and transferred to a 0.2-μm nitrocellulose membrane (catalog no. 10600001; Amersham). After blocking for 1 h at room temperature in 5% nonfat milk, membranes were incubated overnight with primary antibodies at 4 °C. The primary antibodies used were: 1:1,000 rabbit anti-nesprin-2 (catalog no. PA5-78438; Thermo Fisher Scientific); 1:1,000 rabbit anti-LC3B (catalog no. 2775; Cell Signaling Technology); 1:1,000 mouse anti-SQSTM1/p62 (catalog no. ab56416;l Abcam); 1:500 rabbit anti-Syne-1 (catalog no. sc-99065; Santa Cruz Biotechnology); 1:2,000 rabbit anti-β-tubulin (catalog no. ab6046; Abcam); 1:2,000 mouse anti-α-tubulin (catalog no. 12G10; DSHB); 1:10,000 mouse anti-actin (catalog no. MAB1501; Sigma-Aldrich); 1:5,000 mouse anti-fibrillarin (catalog no. ab4566; Abcam); 1:1,000 mouse anti-fibrillarin (catalog no. NB300-269; Novus Biologicals); and 1:5,000 rabbit anti-GFP (catalog no. 701; Minotech). After 3 washes in Tris-buffered saline with Tween20 (20 mM Tris, 150 mM NaCl, 0.1% Tween 20), membranes were incubated for 1 h at room temperature in the corresponding secondary mouse and rabbit horseradish peroxidase-conjugated antibodies at 1:10,000 dilution (catalog nos. ab6789 and ab16284, respectively; Abcam). Development of blots was performed with pico and femto supersignal chemiluminescent substrate (catalog nos. 34578 and 34096, respectively; Thermo Fisher Scientific) according to the manufacturer’s instructions. ImageJ (http://rsb.info.nih.gov/ij/) was used to calculate the relative mean pixel intensity after subtracting the background and normalizing to α-tubulin or β-tubulin. Mean values were compared using unpaired *t*-tests. Each assay was repeated at least three times unless otherwise mentioned. We used the Prism software package (GraphPad Software) for the statistical analyses.

### Neuronal and MEF cultures

MEFs were isolated from embryonic day (E) 14.5 mice (mixed sex), treated with 0.5% trypsin for 10 min. Cortices of E16.5 mice (mixed sex) were dissected, treated with 0.5% trypsin for 15 min at 37 °C, with simultaneous mechanical dissociation. After centrifugation, cells were plated on 24-well plates, with 13-mm diameter round coverslips, treated overnight with 1× poly-L-ornithine hydrochloride (catalog no. P2533; Sigma-Aldrich), followed by 2 h of laminin treatment (catalog no. 23017015; Thermo Fisher Scientific) for 5 d. Cells were cultured in neurobasal medium (B-27; Gibco), supplemented with 1× GlutaMAX (catalog no. 35050038; Thermo Fisher Scientific) and penicillin-streptomycin (catalog no. P4333; Sigma-Aldrich) and cultured for 5 d. Cells were treated with 50 nM bafilomycin A1 (catalog no. B1793; Sigma-Aldrich) and/or Earle’s balanced salt solution (catalog no. E3024; Gibco) for 2 h.

### Immunostaining (paraformaldehyde fixation)

Cultured cells (neurons and MEFs) were washed with PBS and fixed for 15 min in 4% paraformaldehyde in PBS. After fixation, cells were washed with PBS and incubated for 1 h in blocking solution containing 10% FCS and 0.2% Triton X-100 in PBS. Cells were then incubated overnight at 4 °C in blocking solution containing primary antibody (1:50): goat anti-lamin B (catalog no. sc-6216; Santa Cruz Biotechnology); rabbit anti-LC3; guinea pig anti-p62/SQSTM1 (catalog no. GP62-C; Progen); rabbit anti-Syne-1 or rabbit anti-fibrillarin (catalog no. ab5821; Abcam). Coverslips were then washed with PBS, followed by secondary antibody staining for 1 h at room temperature at 1:500 dilution: anti-goat Alexa Fluor 555 (catalog no. ab150134; Abcam); anti-rabbit Alexa Fluor 488 (catalog no. ab150073; Abcam); or goat anti-guinea pig Alexa Fluor 647 (catalog no. ab150187; Abcam). VECTASHIELD Vibrance Antifade Mounting Medium with DAPI (catalog no. H-1800; Vector Laboratories) was used to mount coverslips on slides and stain nuclei. Confocal images of fluorescently labeled proteins were captured using a 40× objective lens on an LSM 710 NL multi-photon confocal microscope (ZEISS). Puncta number, size and pixel intensity measurements were performed with ImageJ after background subtraction and threshold setting.

### Immunostaining (methanol fixation)

WT MEFs were grown on Nunc Cell-Culture 12-well dishes (catalog no. 150628; Thermo Fisher Scientific) with glass coverslips (catalog no. 631-0148; VWR), treated as indicated, washed with Dulbecco’s PBS (catalog no. D8537; Sigma-Aldrich) and fixed with 100% methanol (catalog no. 131091; AppliChem) for 15 min at −20 °C. Cells were washed with Dulbecco’s PBS and blocked with 5% FCS (catalog no. 10500064; Thermo Fisher Scientific), 0.3% Triton X-100 (catalog no. 108643; Merck Millipore), 0.3 M glycine (catalog no. A1067; AppliChem) and Dulbecco’s PBS for 1 h. Then, cells were washed with Dulbecco’s PBS and incubated with primary antibodies (1:50): mouse anti-lamin A (catalog no. ab8980; Abcam) and rabbit anti-fibrillarin or rabbit anti-nesprin-2 in 1% bovine serum albumin (BSA) (catalog no. A1391; AppliChem), 0.3% Triton X-100 and Dulbecco’s PBS overnight. Then, cells were washed with Dulbecco’s PBS and incubated with secondary antibodies (1:500): anti-mouse Alexa Fluor 488 (catalog no. ab150105; Abcam) and anti-rabbit Alexa Fluor 647 (catalog no. ab150075; Abcam) in 1% BSA, 0.3% Triton X-100, Dulbecco’s PBS for 1 h before final washing with Dulbecco’s PBS and mounting with VECTASHIELD Vibrance Antifade Mounting Medium with DAPI. Fluorescent images were acquired using a 60× objective lens (catalog no. AMEP4694; Invitrogen) of the EVOS FL Auto 2 Cell Imaging System. Images were processed and cropped using ImageJ.

### Statistics and reproducibility

Three biological replicates were performed for each experiment, except for Fig. 1i (two biological replicates), Fig. 2h (four biological replicates) and Fig. 3g and Extended Data Figs. 2e and 5d (five biological replicates). Statistical analyses were performed using Prism. Data distribution was assumed to be normal but this was not formally tested. Mean values were compared using unpaired *t*-tests. The error bars of the R-strip charts indicate the s.d. The bar-whisker plots show all the data; the box indicates the 25th–75th percentile range and the whiskers show the min–max values. The extended data Excel spreadsheet refers to all statistical tests used and the respective *P* values. For nematodes, no statistical method was used to predetermine sample size but our sample sizes are similar to those reported in previous publications using similar procedures^9,18,24,30,32,33^. For mice, G power analysis was performed to determine the minimum number of animals required for the in vitro and in vivo experimental procedures. No data were excluded from the analyses. Worms and cells were distributed to the various groups of all experiments from single pulls. Data collection was not randomized. The investigators were not blinded to allocation during the experiments and/or data collection and outcome assessment or analysis. All experiments with objective measurements (such as microscopy and life span assays) were also performed blinded by other members of the laboratory.

### Ethics statement

Our research complies with all relevant ethical regulations. All mouse experiments were performed according to national and European guidelines for the Care and Use of Laboratory Animals. Protocols were approved by the Foundation for Research and Technology-Hellas (FORTH) Ethics Committee (FEC).

### Reporting summary

Further information on research design is available in the Nature Portfolio Reporting Summary linked to this article.

## Supplementary information

Reporting Summary

## Data Availability

The authors declare that all data supporting the findings of this study are available within the paper and its supplementary information files.

## Source data

Fig. 1 Statistical source data.
Fig. 1 Unprocessed western blots.
Fig. 2 Statistical source data.
Fig. 2 Unprocessed western blots.
Fig. 3 Statistical source data.
Fig. 3 Unprocessed western blots.
Fig. 4 Statistical source data.
Fig. 4 Unprocessed western blots.
Fig. 4 Unprocessed western blots.
Fig. 5 Statistical source data.
Fig. 6 Statistical source data.
Extended Data Fig. 1 Statistical source data.
Extended Data Fig. 2 Statistical source data.
Extended Data Fig. 3 Statistical source data.
Extended Data Fig. 4 Statistical source data.
Extended Data Fig. 4 Unprocessed western blots.
Extended Data Fig. 5 Statistical source data.

## References

[1] Baron O (2017). Stall in canonical autophagy-lysosome pathways prompts nucleophagy-based nuclear breakdown in neurodegeneration. Curr. Biol..

[2] Golden TR (2007). Dramatic age-related changes in nuclear and genome copy number in the nematode *Caenorhabditis elegans*. Aging Cell.

[3] Lans H, Hoeijmakers JHJ (2006). Cell biology: ageing nucleus gets out of shape. Nature.

[4] McGee MD (2011). Loss of intestinal nuclei and intestinal integrity in aging *C. elegans*. Aging Cell.

[5] Scaffidi P, Misteli T (2005). Reversal of the cellular phenotype in the premature aging disease Hutchinson–Gilford progeria syndrome. Nat. Med..

[6] Schreiber KH, Kennedy BK (2013). When lamins go bad: nuclear structure and disease. Cell.

[7] Buchwalter A, Hetzer MW (2017). Nucleolar expansion and elevated protein translation in premature aging. Nat. Commun..

[8] Lam YW, Trinkle-Mulcahy L (2015). New insights into nucleolar structure and function. F1000prime Rep..

[9] Tiku V (2017). Small nucleoli are a cellular hallmark of longevity. Nat. Commun..

[10] Lee C-W (2020). Selective autophagy degrades nuclear pore complexes. Nat. Cell Biol..

[11] Papandreou M-E, Tavernarakis N (2019). Nucleophagy: from homeostasis to disease. Cell Death Differ..

[12] Zhang Q (2007). Nesprin-1 and -2 are involved in the pathogenesis of Emery Dreifuss muscular dystrophy and are critical for nuclear envelope integrity. Hum. Mol. Genet..

[13] Banerjee I (2014). Targeted ablation of nesprin 1 and nesprin 2 from murine myocardium results in cardiomyopathy, altered nuclear morphology and inhibition of the biomechanical gene response. PLoS Genet..

[14] Starr DA, Han M (2002). Role of ANC-1 in tethering nuclei to the actin cytoskeleton. Science.

[15] Dou Z (2015). Autophagy mediates degradation of nuclear lamina. Nature.

[16] Mroß C (2018). Depletion of nesprin-2 is associated with an embryonic lethal phenotype in mice. Nucleus.

[17] Mijaljica D, Prescott M, Devenish RJ (2012). A late form of nucleophagy in *Saccharomyces cerevisiae*. PLoS ONE.

[18] Hao H (2021). The nesprin-1/-2 ortholog ANC-1 regulates organelle positioning in *C. elegans* independently from its KASH or actin-binding domains. eLife.

[19] Tian Y (2010). *C. elegans* screen identifies autophagy genes specific to multicellular organisms. Cell.

[20] Hansen M (2008). A role for autophagy in the extension of lifespan by dietary restriction in *C. elegans*. PLoS Genet..

[21] Kenyon C (2011). The first long-lived mutants: discovery of the insulin/IGF-1 pathway for ageing. Philos. Trans. R. Soc. Lond. B Biol. Sci..

[22] Meléndez A (2003). Autophagy genes are essential for dauer development and life-span extension in *C. elegans*. Science.

[23] Lunkes A, Mandel JL (1997). Polyglutamines, nuclear inclusions and neurodegeneration. Nat. Med..

[24] Jia K, Hart AC, Levine B (2007). Autophagy genes protect against disease caused by polyglutamine expansion proteins in *Caenorhabditis elegans*. Autophagy.

[25] Ravikumar B (2004). Inhibition of mTOR induces autophagy and reduces toxicity of polyglutamine expansions in fly and mouse models of Huntington disease. Nat. Genet..

[26] Tiku V, Antebi A (2018). Nucleolar function in lifespan regulation. Trends Cell Biol..

[27] Lee LW, Lee C-C, Huang C-R, Lo SJ (2012). The nucleolus of *Caenorhabditis elegans*. J. Biomed. Biotechnol..

[28] Guo B (2014). Genome-wide screen identifies signaling pathways that regulate autophagy during *Caenorhabditis elegans* development. EMBO Rep..

[29] Muñoz G (2020). Early detection of lysosomal diseases by screening of cases of idiopathic splenomegaly and/or thrombocytopenia with a next-generation sequencing gene panel. JIMD Rep..

[30] Yi Y-H (2015). A genetic cascade of let-7-ncl-1-fib-1 modulates nucleolar size and rRNA pool in *Caenorhabditis elegans*. PLoS Genet..

[31] Monsivais D, Peng J, Kang Y, Matzuk MM (2019). Activin-like kinase 5 (ALK5) inactivation in the mouse uterus results in metastatic endometrial carcinoma. Proc. Natl Acad. Sci. USA.

[32] Jenzer C (2019). Autophagy mediates phosphatidylserine exposure and phagosome degradation during apoptosis through specific functions of GABARAP/LGG-1 and LC3/LGG-2. Autophagy.

[33] Ahmed S, Hodgkin J (2000). MRT-2 checkpoint protein is required for germline immortality and telomere replication in *C. elegans*. Nature.

[34] Kirkwood TBL (2005). Understanding the odd science of aging. Cell.

[35] Ahmed S (2006). Uncoupling of pathways that promote postmitotic life span and apoptosis from replicative immortality of *Caenorhabditis elegans* germ cells. Aging Cell.

[36] Engqvist H (2018). Transcriptomic and genomic profiling of early-stage ovarian carcinomas associated with histotype and overall survival. Oncotarget.

[37] Drenos F, Kirkwood TBL (2005). Modelling the disposable soma theory of ageing. Mech. Ageing Dev..

[38] Kirkwood TB (1987). Immortality of the germ-line versus disposability of the soma. Basic Life Sci..

