# Supplementary material for: Nucleophagy delays aging and preserves germline immortality
Source: Nat Aging. 2022 Dec 23;3(1):34–46. doi: 10.1038/s43587-022-00327-4 (PMC10154226; doi:10.1038/s43587-022-00327-4)
Supplement: Supplementary file 1 — Reporting Summary [file 43587_2022_327_MOESM1_ESM.pdf]

nature portfolio

Corresponding author(s): Nektarios Tavernarakis

Last updated by author(s): Nov 2, 2022

Reporting Summary

Nature Portfolio wishes to improve the reproducibility of the work that we publish. This form provides structure for consistency and transparency in reporting. For further information on Nature Portfolio policies, see our Editorial Policies and the Editorial Policy Checklist.

Statistics

For all statistical analyses, confirm that the following items are present in the figure legend, table legend, main text, or Methods section.

n/a	Confirmed	
☐	☒	The exact sample size (*n*) for each experimental group/condition, given as a discrete number and unit of measurement
☐	☒	A statement on whether measurements were taken from distinct samples or whether the same sample was measured repeatedly
☐	☒	The statistical test(s) used AND whether they are one- or two-sided<br>*Only common tests should be described solely by name; describe more complex techniques in the Methods section.*
☒	☐	A description of all covariates tested
☐	☒	A description of any assumptions or corrections, such as tests of normality and adjustment for multiple comparisons
☐	☒	A full description of the statistical parameters including central tendency (e.g. means) or other basic estimates (e.g. regression coefficient) AND variation (e.g. standard deviation) or associated estimates of uncertainty (e.g. confidence intervals)
☐	☒	For null hypothesis testing, the test statistic (e.g. *F*, *t*, *r*) with confidence intervals, effect sizes, degrees of freedom and *P* value noted<br>*Give P values as exact values whenever suitable.*
☒	☐	For Bayesian analysis, information on the choice of priors and Markov chain Monte Carlo settings
☒	☐	For hierarchical and complex designs, identification of the appropriate level for tests and full reporting of outcomes
☒	☐	Estimates of effect sizes (e.g. Cohen's *d*, Pearson's *r*), indicating how they were calculated

Our web collection on statistics for biologists contains articles on many of the points above.

Software and code

Policy information about availability of computer code

Data collection	ZEN 2012 SP1 (black edition) Version 8.1.5.484 (Zeiss LSM 710)<br>Image LabTM Touch Software Version 2.0.0.27 (ChemiDoc Imaging System)<br>CFX Manager Software Version 3.1 (CFX Real-Time PCR Detection System)<br>DiamondScope 2.0.883.0 (EVOS Invitrogen FL Auto 2.0 Imaging System)
Data analysis	ImageJ 1.53f51 bundled with 64-bit Java 1.8.0_66 (image processing/analysis)<br>GraphPad Prism 8.0 (statistical analysis)

For manuscripts utilizing custom algorithms or software that are central to the research but not yet described in published literature, software must be made available to editors and reviewers. We strongly encourage code deposition in a community repository (e.g. GitHub). See the Nature Portfolio guidelines for submitting code & software for further information.

Data

Policy information about availability of data

All manuscripts must include a data availability statement. This statement should provide the following information, where applicable:

- Accession codes, unique identifiers, or web links for publicly available datasets
- A description of any restrictions on data availability
- For clinical datasets or third party data, please ensure that the statement adheres to our policy

The authors declare that all data supporting the findings of this study are available within the paper and its supplementary information files.

Human research participants

Policy information about studies involving human research participants and Sex and Gender in Research.

Reporting on sex and gender	n/a
Population characteristics	n/a
Recruitment	n/a
Ethics oversight	n/a

Note that full information on the approval of the study protocol must also be provided in the manuscript.

Field-specific reporting

Please select the one below that is the best fit for your research. If you are not sure, read the appropriate sections before making your selection.

☒ Life sciences ☐ Behavioural & social sciences ☐ Ecological, evolutionary & environmental sciences

For a reference copy of the document with all sections, see nature.com/documents/nr-reporting-summary-flat.pdf

Life sciences study design

All studies must disclose on these points even when the disclosure is negative.

Sample size	Mouse experiments used all available animals of the particular genotype and age.<br>C. elegans lifespan experiments used more than 150 animals per individual experiment, which exceeds the typical standard in the field.<br>Fluorescence imaging experiments were replicated three times, with as many animals/cells as could reasonably be analyzed by the analysis methods available.<br>Maximum available samples were used for all experiments.
Data exclusions	No data exclusions.
Replication	Multiple replicates were performed for all experiments as mentioned in methods and figure legends.
Randomization	Several strains used in this study and animals were identified and distributed in experimental groups based on specific fluorescent reporter expression and/or phenotype.
Blinding	All experiments with objective measurements (such as microscopy and lifespan assays) were also performed blinded by other members of the laboratory.

Reporting for specific materials, systems and methods

We require information from authors about some types of materials, experimental systems and methods used in many studies. Here, indicate whether each material, system or method listed is relevant to your study. If you are not sure if a list item applies to your research, read the appropriate section before selecting a response.

Materials & experimental systems

n/a	Involved in the study
☐	☒ Antibodies
☐	☒ Eukaryotic cell lines
☒	☐ Palaeontology and archaeology
☐	☒ Animals and other organisms
☒	☐ Clinical data
☒	☐ Dual use research of concern

Methods

n/a	Involved in the study
☒	☐ ChIP-seq
☒	☐ Flow cytometry
☒	☐ MRI-based neuroimaging

Antibodies

Antibodies used

Western blot - Primary:
Mouse anti-α-Tubulin, DSHB, #12G10, RRID: AB_1157911, 1:2000.
Mouse anti-Actin, Clone C4, SIGMA, #MAB1501, RRID: AB_2223041, 1:10000.
Mouse anti-Fibrillarin (38F3), Abcam, #ab4566, RRID: AB_304523, 1:5000.
Mouse anti-Fibrillarin (38F3), Novus Biologicals, #NB300-269, RRID: AB_523649, 1:1000.
Mouse anti-SQSTM1/p62, Abcam, #ab56416, RRID: AB_945626, 1:1000.
Rabbit anti-β-Tubulin, Abcam, #ab6046, RRID: AB_2210370, 1:2000.
Rabbit anti-GFP, Minotech, #701, https://minotech.gr/index.php/products/anti-hrcn, 1:5000.
Rabbit anti-LC3B, Cell Signalling, #2775, RRID: AB_915950, 1:1000.
Rabbit anti-Nesprin 2, Invitrogen, #PA5-78438, RRID: AB_2736204, 1:1000.
Rabbit anti-Syne-1 (H-100), Santa Cruz Technology, #sc-99065, RRID: AB_2240334, 1:500.
Western blot - Secondary:
Donkey anti-Rabbit HRP, Abcam, #ab16284, RRID: AB_955387, 1:10000.
Goat anti-Mouse HRP, Abcam, #ab6789, RRID: AB_955439, 1:10000.
Immunostaining (PFA fixation) - Primary:
Goat anti-Lamin B (M-20), Santa Cruz Technology, #sc-6216, RRID: AB_648156, 1:50.
Guinea pig anti-p62/SQSTM1, Progen, #GP62-C, RRID: AB_2687531, 1:50.
Rabbit anti-Fibrillarin, Abcam, #ab5821, RRID: AB_2105785, 1:50.
Rabbit anti-LC3B, Cell Signalling, #2775, RRID: AB_915950, 1:50.
Rabbit anti-Syne-1 (H-100), Santa Cruz Technology, #sc-99065, RRID: AB_2240334, 1:50.
Immunostaining (PFA fixation) - Secondary:
Donkey anti-Goat Alexa Fluor 555, Abcam, #ab150134, RRID: AB_2715537, 1:500.
Donkey anti-Rabbit Alexa Fluor 488, Abcam, #ab150073, RRID: AB_2636877, 1:500.
Goat anti-Guinea pig, Alexa Fluor 647, Abcam, #ab150187, RRID: AB_2827756, 1:500.
Immunostaining (methanol fixation) - Primary:
Mouse anti-Lamin A (133A2), Abcam, #ab8980, RRID: AB_306909, 1:50.
Rabbit anti-Fibrillarin, Abcam, #ab5821, RRID: AB_2105785, 1:50.
Rabbit anti-Nesprin 2, Invitrogen, #PA5-78438, RRID: AB_2736204, 1:50.
Immunostaining (methanol fixation) - Secondary:
Donkey anti-Mouse Alexa Fluor 488, Abcam, #ab150105, RRID: AB_2732856, 1:500.
Donkey anti-Rabbit Alexa Fluor 647, Abcam, #ab150075, RRID: AB_2752244, 1:500.

Validation

All the antibodies used in this study are commercially available and validation were performed by the manufacturers and supported by the publications indicated in the manufacturers' websites.

Eukaryotic cell lines

Policy information about cell lines and Sex and Gender in Research

Cell line source(s)

Mouse cell lines and C. elegans strains used in the paper are described in the Methods section.

Authentication

Relevant information is provided in the Methods section.

Mycoplasma contamination

n/a

Commonly misidentified lines
(See ICLAC register)

n/a

Animals and other research organisms

Policy information about studies involving animals; ARRIVE guidelines recommended for reporting animal research, and Sex and Gender in Research

Laboratory animals

Mus musculus: All mice used were of C57BL/6 genetic background.
Caenorhabditis elegans:
N2: wild type Bristol isolate
AM141: rmIs133 [unc-54p::Q40::YFP]

CB1370: daf-2(e1370)
CB3335: anc-1(e1802)
CB3339: anc-1(e1753)
CB3388: ncl-1(e1865)
CB3440: anc-1(e1873)
CF2218: ncl-1(e1942)
COP262: knuSi221 [fib-1p::fib-1(genomic)::eGFP::fib-1 3' UTR + unc-119(+)]
CU1546: smIs34 [ced-1p::ced-1::GFP + rol-6(su1006)]
DA2123: adIs2122 [lgg-1p::GFP::lgg-1 + rol-6(su1006)]
HZ589: him-5(e1490); bpIs151 [sqst-1p::sqst-1::GFP + unc-76(+)]
LW697: ccIs4810 [(pJKL380.4) lmn-1p::lmn-1::GFP::lmn-1 3'utr + (pMH86) dpy-20(+)]
MAH14: daf-2(e1370); adIs2122 [lgg-1::GFP + rol-6(su1006)]
OD95: unc-119(ed3); ltIs37 [pie-1p::mCherry::his-58 + unc-119(+)]; ltIs38 [pie-1p::GFP::PH(PLC1delta1) + unc-119(+)]
VC893: atg-18(gk378)
DA465: eat-2(ad465)
lgg-2(tm5755)
bpIs168 [pnfya-1DFCP1::GFP; unc-76(+)]
atg-2(bp576); bpIs168 [pnfya-1DFCP1::GFP; unc-76(+)]
IR593 N2; Ex[ife-2p::ife-2::GFP + rol-6(su1006)]
IR2969: yc72 [mKate2::anc-1b]; adIs2122 [lgg-1p::GFP::lgg-1 + rol-6(su1006)]
IR2970: yc72 [mKate2::anc-1b]; knuSi221 [fib-1p::fib-1(genomic)::eGFP::fib-1 3' UTR + unc-119(+)]
Caenorhabditis elegans hermaphrodites at day 1 of adulthood grown at 20°C were used unless otherwise specified. All mice were maintained in a pathogen-free environment and housed in clear shoebox cages, in groups of five animals per cage with constant temperature and humidity and a 12h light/12h dark cycle. Apart from the ovarian anatomical studies, all mice used where male of C57BL/6 or Nesprin 2-/- genetic background.

Wild animals	No wild animals were used in this study.
Reporting on sex	Caenorhabditis elegans is a hermaphroditic species and hermaphrodite animals were used for experiments. Sex based analyses were not performed. No sex-specific reporting is included. For mouse experiments, apart from the ovarian anatomical studies, all mice used where male of C57BL/6 or Nesprin 2-/- genetic background.
Field-collected samples	No field-collected animals were used in this study.
Ethics oversight	All mouse experiments were performed according to National and European guidelines for the Care and Use of Laboratory Animals. Protocols were approved by the Foundation for Research and Technology-Hellas (FORTH) Ethics Committee (FEC). No ethical approval is required for the Caenorhabditis elegans studies.

Note that full information on the approval of the study protocol must also be provided in the manuscript.
