# Supplementary figures and images for: Nucleophagy delays aging and preserves germline immortality
Source: Nat Aging. 2022 Dec 23;3(1):34–46. doi: 10.1038/s43587-022-00327-4 (PMC10154226; doi:10.1038/s43587-022-00327-4)

**Fig. 1i**

Nesprin 2  $\epsilon 1$

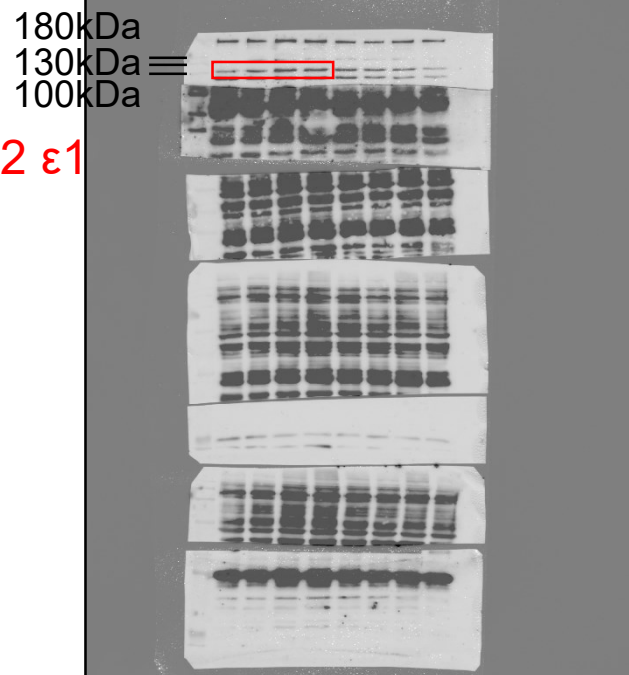

Actin

48kDa —  
35kDa —

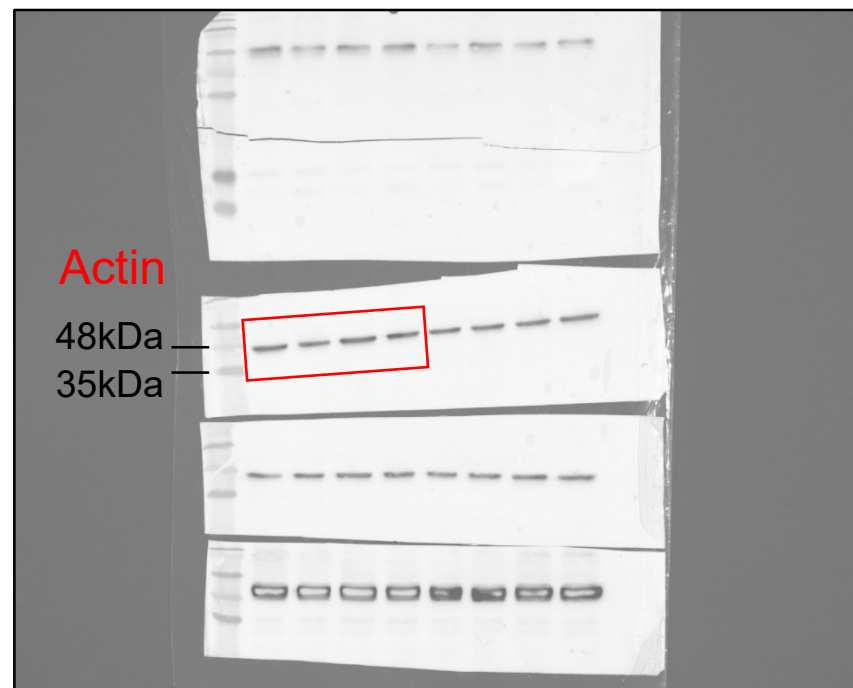

p62

63kDa —  
48kDa —

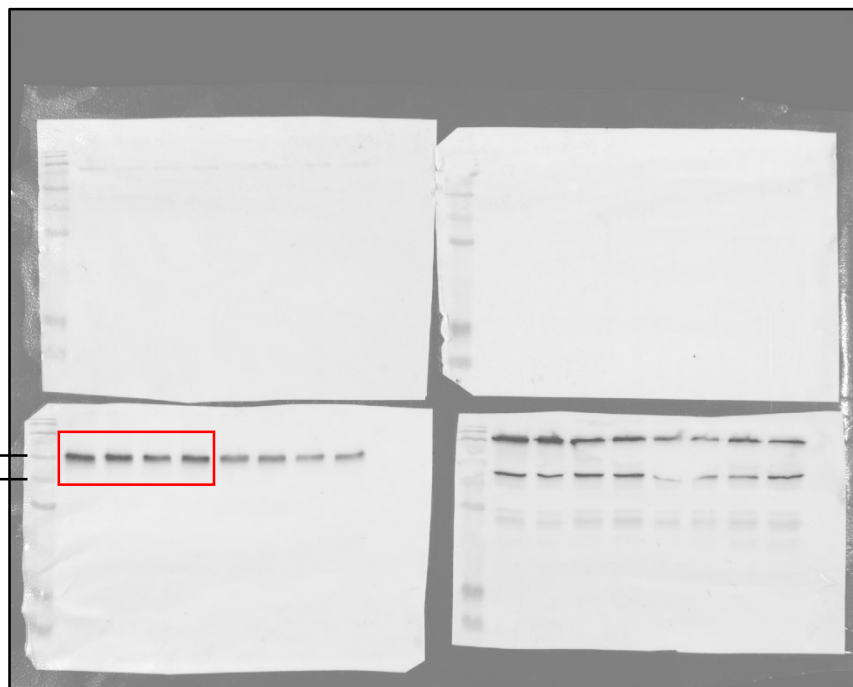

LC3

17kDa —  
10kDa —

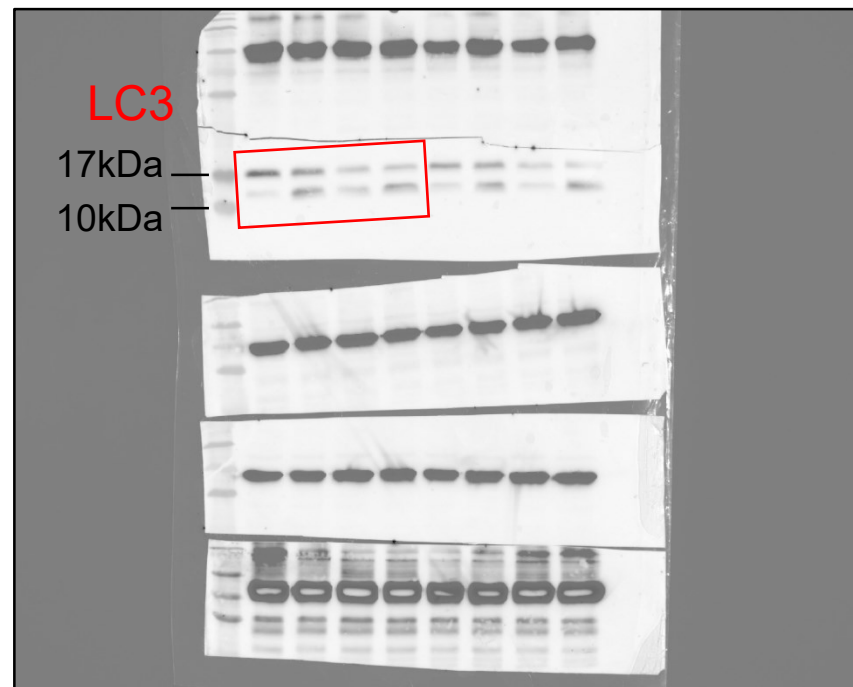

Supplement: Fig. 1 — Unprocessed western blots. [file 43587_2022_327_MOESM3_ESM.pdf]

# Fig. 2g

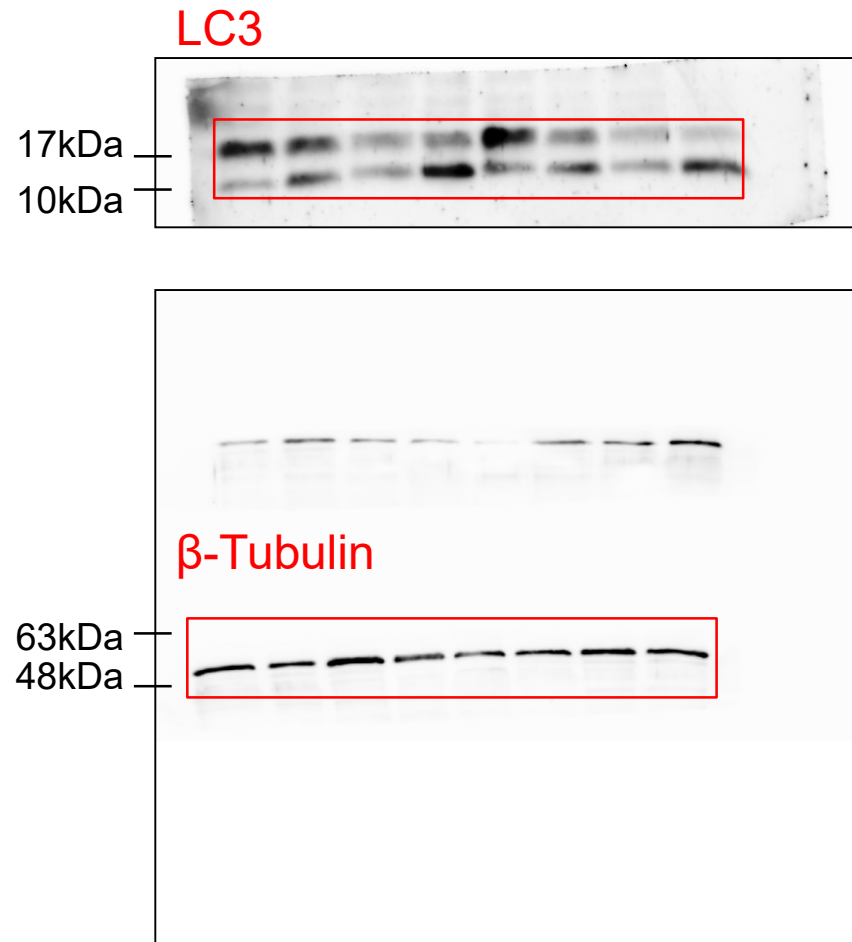

Supplement: Fig. 2 — Unprocessed western blots. [file 43587_2022_327_MOESM5_ESM.pdf]

# Fig. 3d

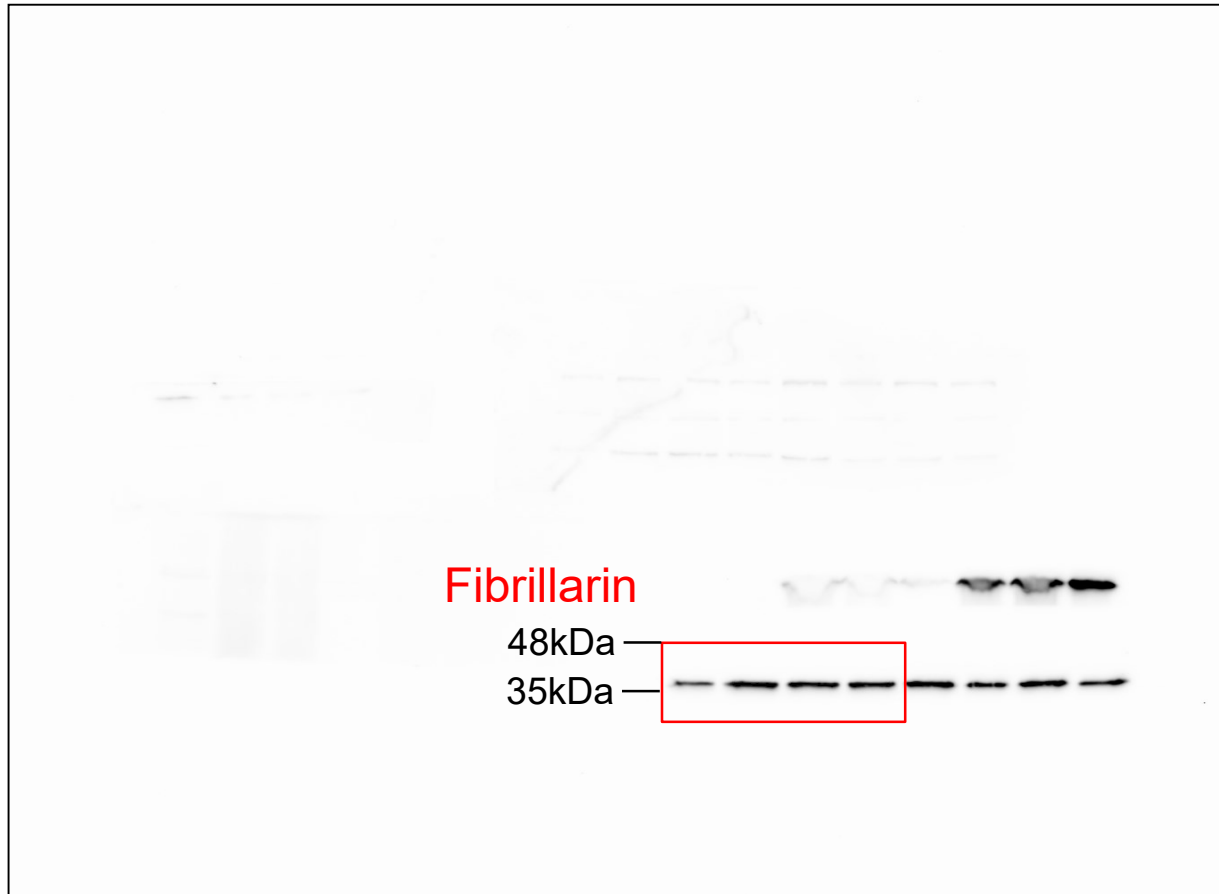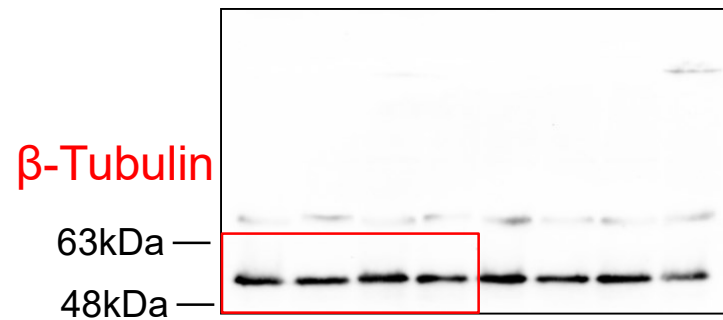

Supplement: Fig. 3 — Unprocessed western blots. [file 43587_2022_327_MOESM7_ESM.pdf]

**Fig. 4b**

GFP (FIB-1::GFP)

75kDa  
63kDa  
48kDa  
35kDa

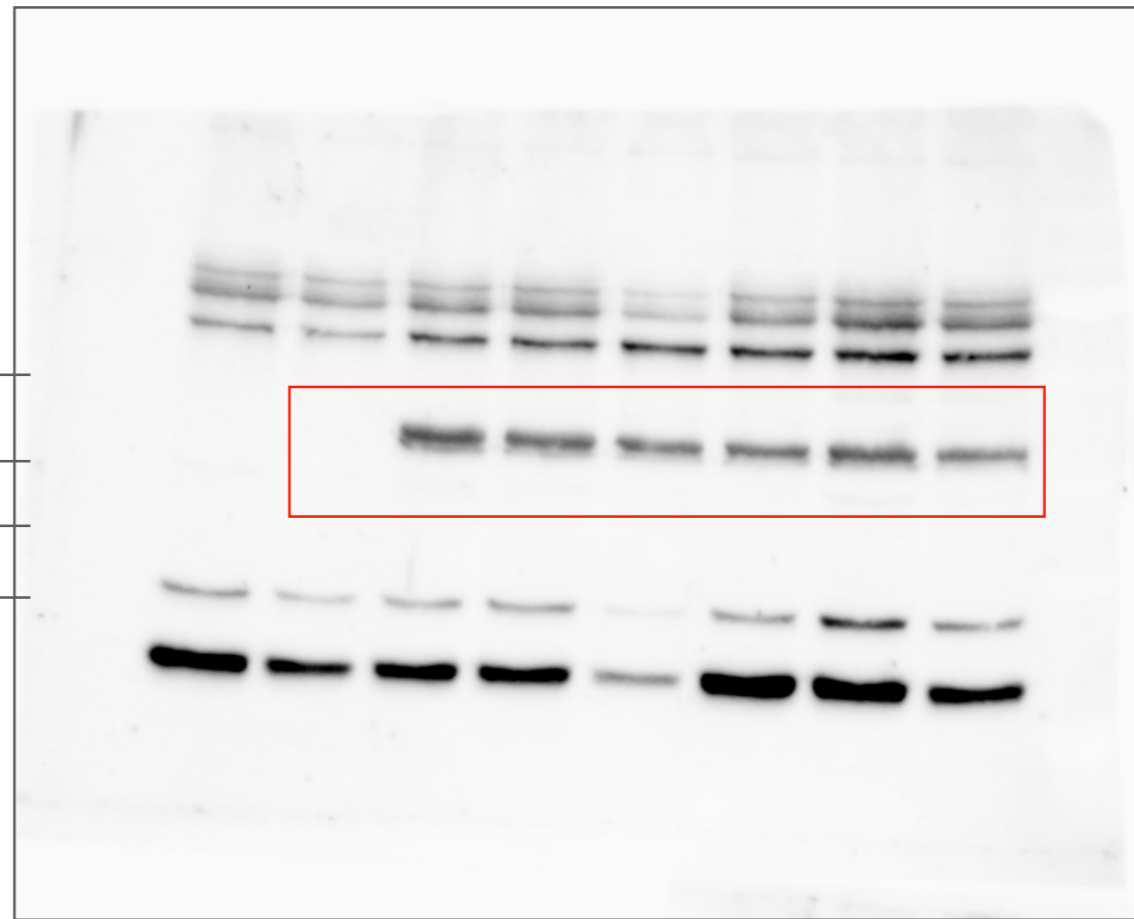

$\alpha$ -Tubulin

63kDa  
48kDa

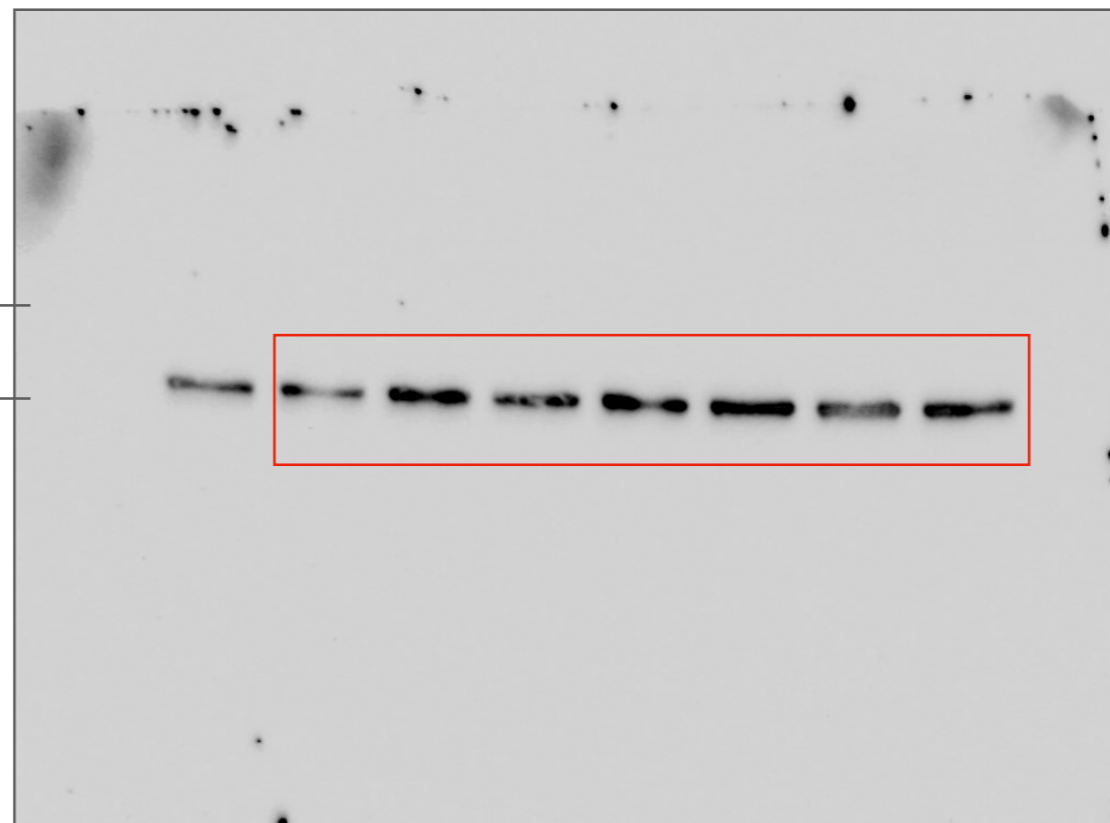

Supplement: Fig. 4 — Unprocessed western blots. [file 43587_2022_327_MOESM9_ESM.pdf]

**Fig. 4c**

Fibrillarin (FIB-1::GFP)

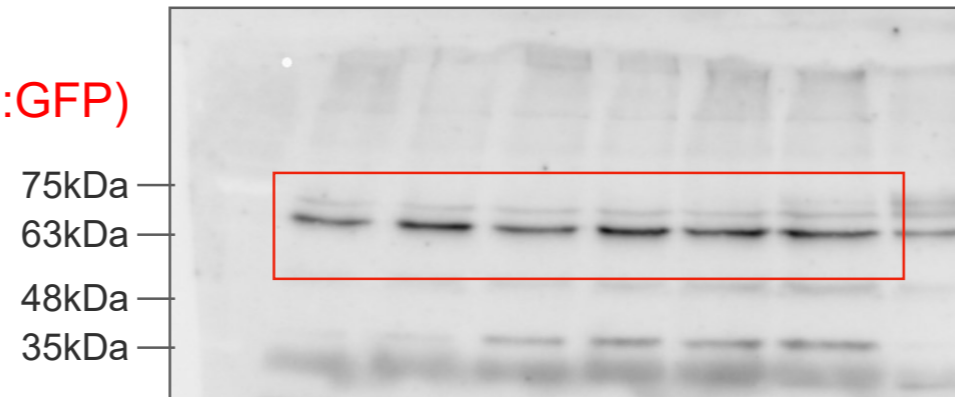

$\alpha$ -Tubulin

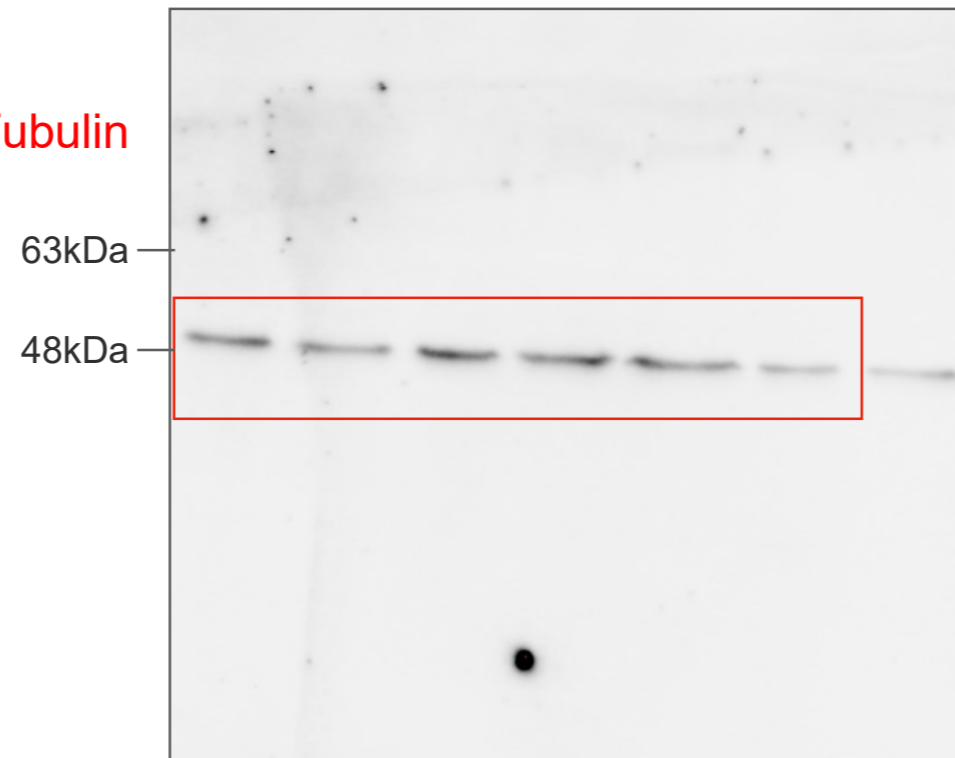

Supplement: Fig. 4 — Unprocessed western blots. [file 43587_2022_327_MOESM10_ESM.pdf]

# ED Fig. 4d

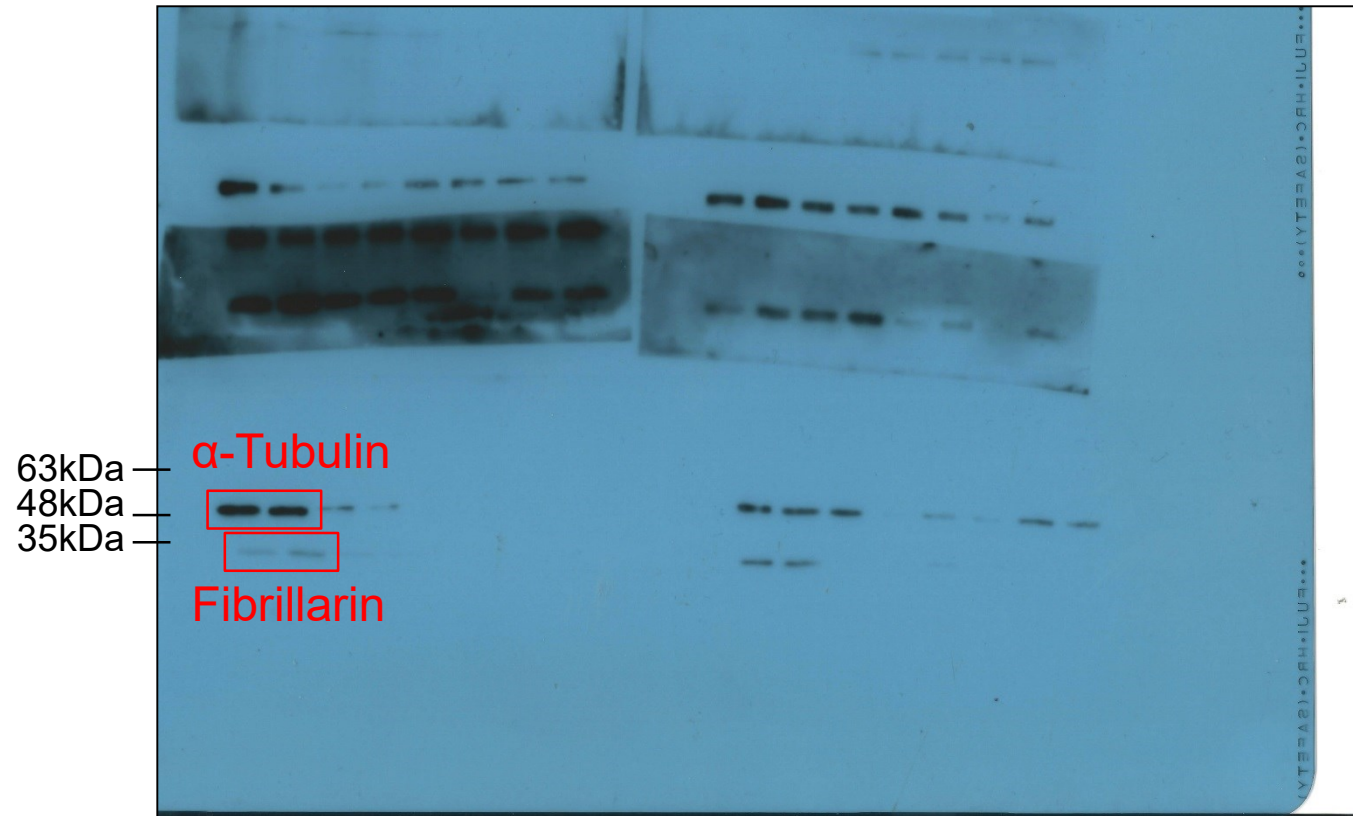

Supplement: Extended Data Fig. 4 — Unprocessed western blots. [file 43587_2022_327_MOESM17_ESM.pdf]
